# Microbial survey of the mummies from the Capuchin Catacombs of Palermo, Italy: biodeterioration risk and contamination of the indoor air

**DOI:** 10.1111/1574-6941.12165

**Published:** 2013-07-09

**Authors:** Guadalupe Piñar, Dario Piombino-Mascali, Frank Maixner, Albert Zink, Katja Sterflinger

**Affiliations:** 1 Department of Biotechnology VIBT-Vienna Institute of BioTechnology, University of Natural Resources and Life SciencesVienna, Austria; 2 EURAC-Institute for Mummies and the IcemanBolzano, Italy

**Keywords:** microbial communities, indoor air quality, degradation, discoloration, DGGE, sequencing analyses

## Abstract

The Capuchin Catacombs of Palermo contain over 1800 preserved bodies dating from the 16th to 20th centuries AD and showing evidence of biodeterioration. An extensive microbiological and molecular investigation was recently performed. Samples were taken from skin, muscle, hair, bone, stuffing materials, clothes, and surrounding walls as well as from the indoor air. In this study, we witnessed that the different degradation phenomena observed on the variety of materials located at the Capuchin Catacombs of Palermo are biological in origin. Molecular techniques showed the dominance of halophilic species of the domains *Bacteria* and *Archaea* on the walls and – as a result of salt emanating from the walls – on the mummies themselves. Nevertheless, specialized microorganisms belonging to taxa well-known for their cellulolytic and proteolytic activities were detected on clothes and stuffing material, and on skin, muscle, hair, and bone, respectively. This specialized microbiota is threatening the conservation of the mummies themselves. Additionally, sequences related to the human skin microbiome and to some pathogenic *Bacteria* (order *Clostridiales*) and fungi (genus *Phialosimplex*) were identified on samples derived from the mummies. Furthermore, a phosphate-reducing fungus, *Penicillium radicum,* was detected on bone. Finally, the high concentration of airborne fungal spores is not conducive to the conservation of the human remains and is posing a potential health risk for visitors.

## Introduction

Mummies have been widely investigated by molecular techniques. These studies have led to a new branch of science called paleomicrobiology (Drancourt & Raoult, 2005). A particularly interesting sector within this field is the study of ancient microorganisms, bacteria, filamentous fungi, yeast, algae, protozoans, and viruses. The analyses of ancient microorganisms DNA in ancient human remains are contributing to the understanding of various issues such as the spread of diseases (Zink *et al*., 2002, 2003; Fernandez, 2012), the mummification processes (Rollo *et al*., 2000), and the effect of diet and hygiene conditions on historical human populations (Cano *et al*., 2000). Nevertheless, this study is not focused on ancient microorganisms, but on those opportunistic microorganisms able to colonize and deteriorate the preservation of ancient bodies. There are several well-known examples showing the colonization of preserved bodies by opportunistic fungi, such as the case of the restoration of the body of Ramses II, performed in Paris in 1976–77. The mummy showed a dense fungal population with species belonging to the genera *Aspergillus* and *Penicillium* (Mouchaca, 1985). Aspergilli also dominated the microbial communities of the air and dust of the Egyptian mummy chamber at the Baroda Museum in India (Arya *et al*., 2001). Additionally, saprophytic fungi belonging to the genera *Monilia*, *Penicillium*, *Alternaria*, *Aspergillus*, *Rhizopus,* and *Chrysosporium* as well as saprophytic bacteria of the genus *Bacillus* were isolated from a mummy from the collection of the Archaeological Museum in Zagreb, Croatia (Čavka *et al*., 2010). Furthermore, one of the most obvious decomposition phenomena seen in histological sections of archaeological bone is focal destruction or ‘tunneling’, a loss of structural integrity which can destroy the original structure of the bone. These tunnels are presumed to be of fungal origin (Grupe & Dreses-Werringloer, 1993).

**Figure 1:**
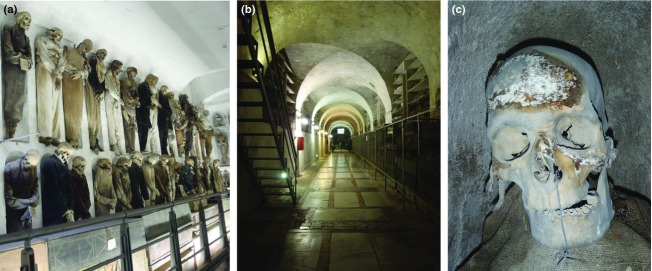
(a) Mummies displayed along the walls of the Capuchin Catacombs of Palermo, Italy. (b) Ground view of a corridor of the Catacombs. (c) Mummy heavily contaminated with molds (pictures: Sterflinger).

In this study, we intended to gain an initial insight into the microorganisms involved in the phenomena of biodeterioration observed on the human remains and related materials, in the Capuchin Catacombs of Palermo, Italy. At the end of the 16th century, the Catacombs were constructed as a burial site for deceased friars. Over time, huge subterranean corridors were carved out of a massive deposit of tuff that underlies the Capuchin Church and the Convent. The first mummified bodies were placed there in 1599, and the last ones in the early 20th century (Piombino-Mascali *et al*., 2010). Today, the Catacombs form an impressive site where over eighteen hundred bodies, many of which still retain soft tissue, are displayed along the sides of the corridors or stored in coffins (Fig. 1a and b). The Catacomb mummies of Palermo are mainly the result of a spontaneously-enhanced preservation mechanism (Aufderheide, 2003). Shortly after death, bodies were taken to special preparation rooms, laid on terracotta pipes conceived to allow draining of the body fluids, and promote spontaneous desiccation of the cadavers. The rooms were then sealed for about a year, after which time the corpses were exposed to the air, washed with vinegar, and dressed. Some bodies were also preserved with anthropogenic methods, such as dipping into lime or arterial injections with special chemicals (Piombino-Mascali *et al*., 2010).

**Figure 2:**
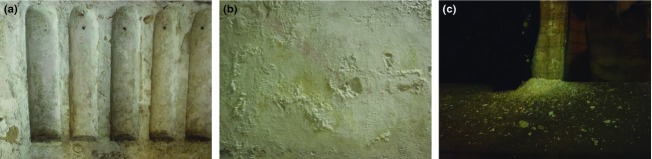
(a) Pink discoloration of the wall caused by halophilic *Bacteria*. (b) Salt crusts of sodium chloride on the walls. (c) Detached salt contaminating other materials (pictures: Sterflinger).

Within the framework of the ‘Sicily Mummy Project’ (Piombino-Mascali *et al*., 2011), a research project aimed at studying and preserving these remains, we were able to conduct an in-depth microbiological and molecular investigation to reveal the microbiota involved in the biodeterioration observed in the Capuchin Catacombs of Palermo. Several problems were noted: Firstly, the poor indoor air quality was unsuitable for the safe conservation of the human remains and could pose a health risk to visitors. Secondly, the walls of the Catacombs were showing an intriguing extensive rosy discoloration possibly with a biological origin (Fig. 2a). In addition, the environmental conditions in the Catacombs allowed the formation of salt deposits over the walls (Fig. 2b), producing salt cracks detaching and contaminating the surfaces of other materials (Fig. 2c). Thirdly, we observed the moldy appearance of many mummies, especially those located in corridors with high humidity (Fig. 1c). Finally, besides the superficial salt contamination, we investigated whether specialized microorganisms, with potential degradation activities, were present on, and inside, the mummy materials. A sampling campaign was performed at the Catacombs, including the air, the surrounding walls, and different materials from the human remains located in the Capuchin crypt, with the goal of answering these open questions. Molecular techniques, including direct DNA extraction from all different sampled materials, PCR amplification using specific primers for *Bacteria*, *Archaea*, and fungi, and further denaturing gradient gel electrophoresis (DGGE)-fingerprinting and sequencing, enabled the detection and characterization of the microbial communities specifically colonizing all types of materials.

## Materials and methods

### Sampling and cultivation of fungi

Air samples from each corridor were collected in triplicate using the air sampler Sartorius MD8 with an airflow of 30 L min^−1^ and using Petri dishes with 2% MEA and DG18 (Merck, Austria). The total volume of air filtered was 100 L. Incubation was 7 days at 22 °C.

Samples from different materials of the mummies including skin, muscle, hair, bones, clothes, as well as tow and straw used as stuffing material were taken with sterile scalpels and forceps (Bayha GmbH, Germany) for molecular analyses. These samples were obtained with the restricted permission of the curator (Dr Piombino-Mascali) due to the valuable nature of the objects. Samples derived from the surrounding walls showing the same phenomena (rosy discoloration, salt efflorescences, or purple spots) were taken from all locations within the Catacombs and pooled for molecular analyses.

In addition, noninvasive sampling using DG18 and 2% MEA contact plates (Heipha) was performed on different materials all over the corridors for cultivation of superficial growing fungi.

Fungi collected from the air samples on filters (Sartorious, Germany) were purified by several transfers onto 2% MEA and DG18 (Merck). Pure cultures were identified based on their morphology and sequencing of rDNA (ITSI-5.8S-ITSII).

### DNA extraction

DNA extraction was performed directly from all material samples using the method previously described by Sert & Sterflinger (2010) with the following modifications: pieces of different materials (20–50 mg for mummy material and 50–100 mg for wall material) together with 500 μL extraction buffer I [50 mM Tris-HCl, 150 mM NaCl, 50 mM EDTA, and 0.3% SDS (w v^−1^), pH 8.0] were added to the lyzing matrix E tubes (MP Biomedicals, Illkrich, France). After vortexing, the sample was processed twice in the Fast Prep FP120 Ribolyzer (Thermo Savant; Holbrook) for 40 s at speed 6 (m s^−1^). Between these ribolyzing steps, the sample was incubated at 65 °C for 1 h. After centrifugation for 10 min at 9300 ***g*** (all centrifugation steps at 4 °C), the supernatant was transferred into a new microfuge tube. Further DNA extraction was carried out with 1 : 1 Vol. chloroform/isoamyl alcohol (24 : 1 v v^−1^; Roth). During vortexing, a white interface formed, and after centrifugation for 5 min at 15700 ***g***, the aqueous supernatant was transferred into a new tube. This step was repeated using the same volume (1 : 1 Vol) phenol/chloroform/isoamyl alcohol (25 : 24 : 1, v v^−1^; Roth). Prior to centrifugation after the addition of chloroform and phenol, tubes were incubated at 5 °C for 5 min. After a centrifugation step (5 min at 15 700 ***g***), the supernatant was transferred to a new microfuge tube and further purified using the QIAamp Viral RNA mini kit (Qiagen, Hilden, Germany) following the instructions of the manufacturer. The final elution step was repeated twice with 100 μL of 80 °C preheated ddH2O (Sigma Aldrich, St. Louis). The purified DNA was used directly for PCR amplification.

The concentration and quality of the DNA extracts were assessed using a NanoDrop® ND-1000 Spectrophotometer (peqLab Biotechnologie GmbH, Linz, Austria). The analyses were performed according to the manufacturer's protocol, and the extracted DNA was analyzed in duplicate.

### PCR amplification of extracted DNA

For all PCR reactions, 2X PCR Master Mix from Promega (Vienna, Austria) [50 units mL^−1^ of TaqDNA polymerase supplied in an appropriate reaction buffer (pH 8.5), 400 μM dATP, 400 μM dGTP, 400 μM dCTP, 400 μM dTTP, 3 mM MgCl_2_] was diluted to 1X and 12.5 pmol μL^−1^ of each primer (stock: 50 pmol μL^−1^, VBC-Biotech, Austria) were added. In a total volume of 25 μL, 400 μg mL^−1^ BSA (stock: 20 mg mL^−1^; Roche, Diagnostics GmbH, Germany), and 2.5 μL DNA template were added. PCR was performed in a MJ Research PTC-200 Peltier Thermal Cycle.

For the analysis of fungal sequences, fragments of 450–600 bp in size corresponding to the ITS1, the ITS2 region, and the adjacent 5.8S rRNA gene, were amplified with the primer pair ITS1 and ITS4 (White *et al*., 1990). For DGGE analysis, a nested PCR was performed with the PCR product of the first round as template DNA using the primers ITS1GC with a 37-base GC clamp attached to the 5′ end (Muyzer *et al*., 1993) and ITS2. All reactions were carried out as described by Michaelsen *et al*. (2006).

For the amplification of bacterial 16S rRNA gene sequences, DNA was amplified with the primer pair 341f/985r (Muyzer *et al*., 1993; Heuer *et al*., 1999). For DGGE analysis, 200-bp fragments spanning the hypervariable V3 region of the 16S rRNA gene, were amplified with a nested PCR using the eubacterial-specific primer 341f-GC with a 40-bp GC clamp added to its 5′ end (Muyzer *et al*., 1993) and the universal consensus primer 518r (Neefs *et al*., 1990). PCR conditions were as described by Schabereiter-Gurtner *et al*. (2001).

For the amplification of archaeal 16S rRNA gene sequences, the primer pair ARC344f/ARC915r (Raskin *et al*., 1994) that amplify a PCR fragment of 590 bp was used. For DGGE analysis, *c*. 200-bp fragments of the 16S rRNA gene were amplified with a nested PCR using the archaeal-specific primer ARC344f (Raskin *et al*., 1994) and the universal consensus primer 518r (Neefs *et al*., 1990) with a 40-bp GC clamp added to its 5′ end (Muyzer *et al*., 1993). PCR conditions were as described by Piñar *et al*. (2001). All PCR products were analyzed by electrophoresis in a 2% (w/v) agarose gel.

### Denaturing gradient gel electrophoresis

DGGE was performed as previously described (Muyzer *et al*., 1993) using a D-Code system (Bio-Rad) in × 0.5 TAE (20 mM Tris, 10 mM acetate, 0.5 mM Na_2_EDTA, pH 7.8 with 8% (w v^−1^) acrylamide). Gels were run at a constant temperature of 60 °C with a voltage of 200 V during 3.5 h for *Bacteria* and *Archaea*, and 6 h for fungal fingerprints. The linear chemical gradient of denaturants used in this study [100% denaturing solution contains 7 M urea and 40% (v v^−1^) formamide] are indicated in the legend of figures.

After completion of electrophoresis, gels were stained in a 1 μg mL^−1^ ethidium bromide solution [stock: 10 mg mL^−1^] for 20 min and afterward visualized by a UVP documentation system (Bio-Rad Transilluminator, Universal Hood, Mitsubishi P93D-printer).

### Creation of clone libraries and sequence analysis

To obtain a detailed phylogenetic identification of the microbial community members, clone libraries containing either ITS fungal regions (fungal community) or 16S rRNA gene fragments (bacterial or archaeal communities) were created. For the construction of clone libraries, 2 × 3 μL DNA templates of each sample were amplified in 2 × 50 μL reaction volumes using the following primer pair combinations: for fungal clone libraries, the DNA template was amplified using the primers ITS1/ITS4, as mentioned above. The primer pairs 341f/985r and ARC344f/ARC915r were used for bacterial clone libraries and archaeal clone libraries, respectively. The PCR products were purified using the QIAquick PCR Purification Kit Protocol (Qiagen) and resuspended in ddH2O water.

Purified PCR products were ligated into the pGEM-T easy Vector system (Promega) following the instructions of the manufacturer. The ligation products were transformed into One shot TOP10 cells (Invitrogen). The cells, which allow the identification of recombinants (white colonies), were plated in duplicate with the dilution factor recommended by the manufacturers on an indicator LB medium containing ampicillin (100 μg mL^−1^), streptomycin (25 μg mL^−1^), and X-Gal (5-bromo-4-chloro-3-indolyl-ß-1-galactopyranoside; 0.1 mM) (Sambrook *et al*., 1989).

Fifty clones per each clone library were screened in a DGGE gel and sequenced as described by Schabereiter-Gurtner *et al*. (2001). Comparative sequence analysis was performed by comparing pairwise insert sequences with those available in the public online database NCBI using the blast search program (Altschul *et al*., 1997), and in addition, the most similar sequences were searched in the RDPII database using the SeqMatch tool (Cole *et al*., 2009). The resulting sequences of the bacterial, archaeal, and fungal clones have been deposited at the GenBank: Genetic sequence database at the National Center for Biotechnical Information (NCBI) and the accession numbers are included in Supporting Information, Tables S1a and S1b (*Bacteria*), S2 (*Archaea*), and S3 (fungi).

## Results and discussion

### Indoor air quality at the Catacombs

**Figure 3:**
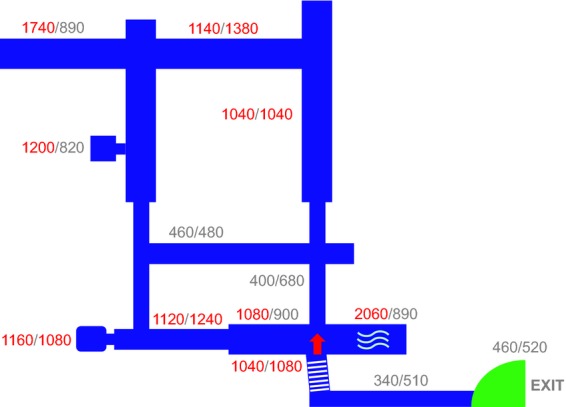
Sketch of the corridors showing the air sampling locations and the corresponding fungal spore concentrations (CFU m^−3^ air). Values show the average of three measurements performed each on 2% MEA and DG18 media, respectively.

Nowadays, it is well known that the quality of indoor air suitable for the safe conservation of exhibited objects of cultural interest is very difficult to assess. Nevertheless, the fewer the contaminants present in the indoor air, the more the confined environment will preserve the exhibited objects (La Genussa *et al*., 2005). In order to investigate the impact of microbial contamination on the indoor air quality at the Catacombs, air samples were collected from different areas of the many corridors and analyzed both quantitatively and qualitatively using Petri dishes with 2% MEA and DG18 media, as described in the section. The fungal spore concentrations (CFU m^−3^ air) recorded using both media are showed in Fig. 3.

Results showed that in some areas of the crypt, the amount of fungal spores present in the air exceeded 2000 spores m^−3^. Medically, this amount must be classified as posing a potential health risk to visitors. Indeed, there are Italian Standards: UNI 10829 (1999) and UNI 10969 (2002) providing the guidelines to choose and control the indoor microclimate in order to correctly keep the artifacts. To date, these guidelines have not been adopted to control the indoor air quality of the Catacombs of Palermo.

### Wall samples

First observations revealed an extensive rosy discoloration of the Catacomb walls (Fig. 2a), which are in direct contact with the surrounding soil. In some areas, water is migrating horizontally into the walls of the Catacombs, carrying soluble salts. Due to changes in physical parameters, salts from the solution precipitated on the exposed surface, creating salt efflorescences (Amoroso & Fassina, 1983), which are dispersed all over the walls (Fig. 2b). The crystallization of salts on the wall of the Catacombs has resulted in a destructive effect. Some salts can crystallize to different hydrates occupying a larger space and producing an additional pressure that finally produces material losses and destruction due to cracking and detachment of the walls. This detached material is accumulating on the surface of both coffins and mummies, producing further contamination of these materials (Fig. 2c). Moreover, the salt efflorescence mimics the conditions found in extreme habitats favoring the proliferation of halotolerant/halophilic microorganisms (Piñar *et al*., 2009).

Three samples, W1 (rosy discolored wall), W2 (salt efflorescence), and PS (wall showing purple stains) collected and pooled from different areas of the walls of the Catacombs were analyzed by culture-independent techniques. All three samples yielded pure DNA extracts with concentrations of 34.21–76.06 ng DNA mg^−1^ of sample. The DNA extracts were amplified by PCR with primers targeting the 16S rRNA gene of *Bacteria* and *Archaea* as well as the ITS regions of fungi. PCR analysis using eubacterial-specific primers showed positive results for all wall samples, whereas archaeal-specific primers showed positive results only for sample W2, indicating the presence of these specialized microorganisms on salt efflorescences. Fungal-specific primers yielded no amplification of the extracted DNAs, confirming the low relative abundance of these microorganisms on the walls (data not shown). This can be explained by the fact that the growth of common hyphomycetes is generally suppressed by high salt stress and only a limited number of fungal species are halotolerant or halophilic (Gunde-Cimerman *et al*., 2009).

**Figure 4:**
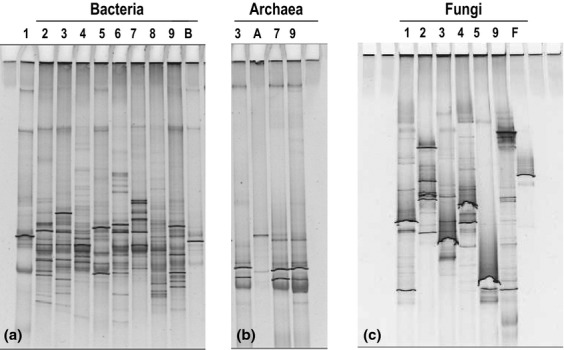
DGGE-fingerprints derived from (a) bacterial, (b) archaeal, and (c) fungal communities colonizing all different material collected at the Capuchin Catacombs. The linear chemical gradient of denaturants used was 25–55% for *Bacteria* and *Archaea*, and 20–50% for fungi. Lane 1: muscle; lane 2: clothes; lane 3: bone; lane 4: hair; lane 5: skin; lane 6: rosy discolored wall; lane 7: salt efflorescence; lane 8: wall with purple stains; lane 9: stuffing material; B: positive control *Bacteria*; A: positive control *Archaea*; F: positive control fungi.

**Figure 5:**
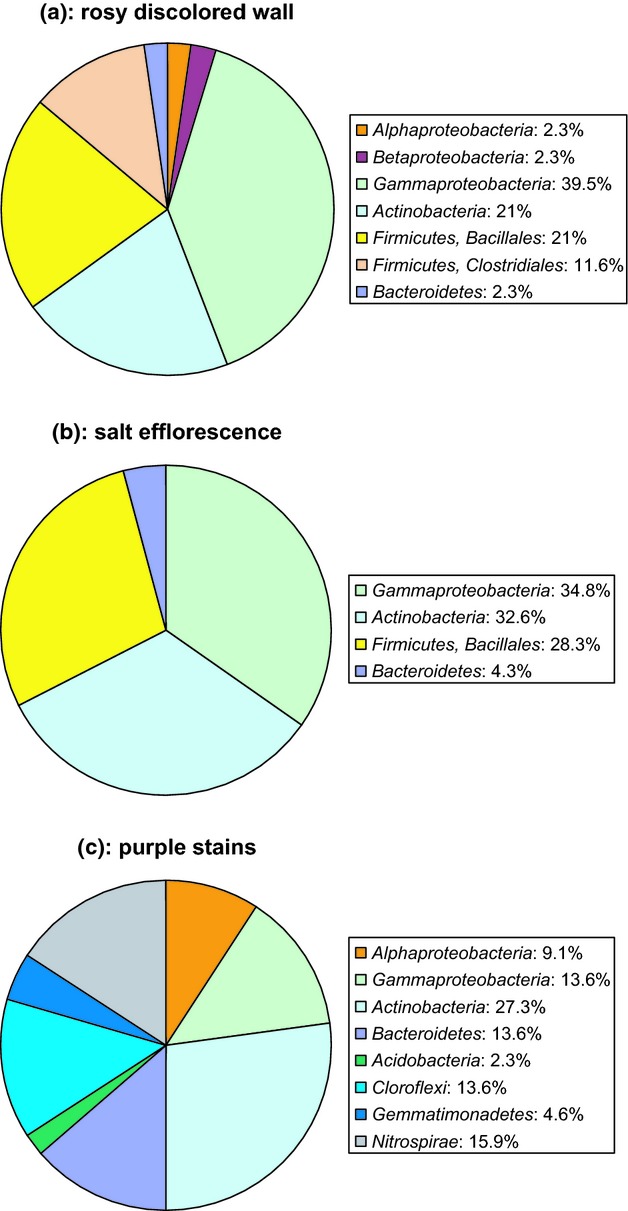
Diagram showing the relative abundance of the bacterial diversity present on wall samples. (a) rosy discolored wall. (b) salt efflorescence. (c) wall with purple stains.

The bacterial and archaeal 16S rRNA gene amplified fragments were further analyzed using DGGE-fingerprints. This technique allowed an estimation of the most abundant organisms inhabiting the walls of the Catacombs and showed the putative differences in microbial composition among different samples. The obtained DGGE-profiles are shown in Fig. 4a (lanes 6, 7, and 8 – *Bacteria*) and in Fig. 4b (lane 7 – *Archaea*). DGGE-fingerprints derived from bacterial sequences of samples W1, W2, and PS revealed complex bacterial communities, whereas those derived from archaeal sequences showed less diversity. To accomplish phylogenetic identification of the bacterial and archaeal communities inhabiting the hypersaline environment presented by the Catacomb walls, clone libraries containing the 16S rRNA gene fragments of these two domains were generated from all samples that yielded positive PCR results. Clones were screened by DGGE and those displaying different fingerprints were grouped. Finally, one representative of each group was selected for sequencing. The obtained sequences were compared with those of known *Bacteria* in the NCBI database. Table S1a shows the phylogenetic affiliations of the selected bacterial clones sequenced from wall samples and their percentages in the clone libraries. The comparative sequence analyses revealed similarity values ranging from 94% to 100% with sequences from the NCBI database. In addition to the best blast hit analysis, most similar sequences were searched in the RDPII database using the SeqMatch match tool. Three main phylogenetic groups were present in all samples: *Proteobacteria* (*Alpha*-, *Beta*- or *Gamma*-classes), *Actinobacteria,* and *Bacteroidetes*. Members of the phylum *Firmicutes* (order *Clostridiales* and/or *Bacillales*) were present on W1 and W2 samples, but absent on the PS sample. However, the last sample showed the highest level of bacterial diversity, and in addition to the three first above-mentioned phyla, members belonging to the *Acidobacteria*, *Chloroflexi*, *Gemmatimonadetes,* and *Nitrospirae* phyla were also detected (Fig. 5 and Table S1a).

On sample W1, taken from an area with an extensive rosy discoloration, members of the *Gammaproteobacteria* (Fig. 5a) dominated on the bacterial community (39.5% of the screened clones) and halotolerant and halophilic species related to the genera *Salinisphaera* (13.9%) and *Halomonas* (13.9%) were detected. In addition, other clones of this class affiliated with cultivated species of the genus *Acinetobacter* (9.3%) and with cultivated species of the genus *Legionella* (2.3%). Members of the *Alpha*- and *Betaproteobacteria* were also detected in this sample, but in a lower proportion (2.3% each), being related to uncultured clones and to sequences related to the genera *Devosia* and *Cupriavidus*, respectively, both previously detected on stone and soil samples (Kumar *et al*., 2008). The phylum *Actinobacteria* accounted for the 21% of screened clones. Surprisingly, most of these sequences showed to be related to *Corynebacterium kroppenstedtii* (11.6%), which was isolated from human clinical material and human sputum (Bernard *et al*., 2002; Tauch *et al*., 2008) and is nowadays known to be a member of the skin microbiome (Microbiome project, Grice *et al*., 2009). Moreover, 4.6% of clones display high sequence similarity to uncultured bacterial clones associated with disease flares in children with atopic dermatitis (Kong *et al*., 2012). The rest of the actinobacterial clones were related to typical stone and soil-inhabiting microorganisms such as *Frankia* sp. (2.3%), and members of the subclass *Rubrobacteridae* (2.3%), a group of very slow-growing and mini-colony-forming soil *Bacteria*, very difficult to be detected using conventional cultivation techniques (Davis *et al*., 2011). Members of the phylum *Firmicutes* were also detected on this sample, belonging to the order *Bacillales* (21%) with sequences related to *Paenibacillus* spp. found in soils, and belonging to the order *Clostridiales* (11.6%) with sequences related to the genus *Clostridium* (Macdonald *et al*., 2011). Finally, 2.3% of the screened clones affiliated with halotolerant members of the phylum *Bacteroidetes*.

Sample W2, taken directly from a salt efflorescence (Fig. 5b), showed the lowest bacterial diversity among the wall samples. The G*ammaproteobacteria* accounted for the 34.8% of the screened clones, and sequences were related to halotolerant and halophilic species related to the genera *Salinisphaera* (2.2%), *Halomonas* (4.3%), and *Idiomarina* (23.9%). In addition, species of the *Luteibacter* genus (4.3%) previously detected in soil were detected. The phylum *Actinobacteria* (32.6%) was represented mainly by species of the genus *Rubrobacter* (28.3%) previously isolated from deteriorated monuments and often associated with a rosy discoloration phenomenon (Laiz *et al*., 2009; Jurado *et al*., 2012). However, some sequences also proved to be related to uncultured clones from the skin microbiome. The other 4.3% of actinobacterial sequences was related to an uncultured bacterial clone detected on mold-colonized water-damaged building materials, indicating a high abundance and variety of *Actinobacteria* in such environments (Schäfer *et al*., 2010). The phylum *Firmicutes* was represented by the order *Bacillales* (28.3%), and most of the sequences affiliated with species of the genus *Sediminibacillus* (15.2%), isolated from hypersaline environments (Wang *et al*., 2009); 6.5% of clones related to this order affiliated with alkaliphilic uncultured clones as well as with cultivated alkaliphilic bacilli. Alkaliphilic *Bacillus* species have important industrial applications due to their ability to produce alkaline enzymes such as protease and cellulase (Nogi *et al*., 2005). In addition, 6.5% of clones were related to an uncultured clone as well as to *Thermoactinomyces sacchari*, a thermophilic *Actinomycete*-like bacterium involved in hypersensitivity pneumonitis (Harvey *et al*., 2001). The *Bacteroidetes* phylum (4.3%) was represented by sequences related to an uncultured bacterium clone found on cellulosic waste containing known cellulose-degrading microorganisms (Field *et al*., 2010).

Sample PS, taken from an area with purple stains, showed the highest bacterial diversity (Fig. 5c). The phylum *Proteobacteria* was represented by members of the *Gammaproteobacteria* (13.6% of screened clones), namely by sequences related to cultured species of the genus *Cellvibrio* (9.1%), with cellulose-degrading activities (Lednická *et al*., 2000) and to uncultured clones (2.3%), and sequences related to *Moraxella sp*. (2.3%). The *Alphaproteobacteria* were also represented (9.1%), namely by sequences related to uncultured clones as well as to cultivated species of the genus *Hyphomicrobium* (6.8%). In addition, 2.3% of clones affiliated with a *Parvularculaceae* bacterium. The phylum *Actinobacteria* dominated on this sample (27.3%), and 11.4% of these clones affiliated with members of the *Rubrobacteridae* and the genus *Rubrobacter*, as observed on sample W2; 11.4% of clones showed to be related with species of the genus *Nocardioides*, previously isolated from aquatic environments (Lee *et al*., 2008), 2.3% with *Mycobacterium* spp. isolated from marine organisms (Izumi *et al*., 2010) and 2.3% with species of the genus *Pseudonocardia*. The *Bacteroidetes* and the *Chloroflexi* phyla accounted each for 13.6% of the screened clones and sequences affiliated with halotolerant species of the *Bacteroidetes* and with uncultured clones of the *Chloroflexi*. The phylum *Nitrospirae* (15.9%) was represented by an uncultured bacterium clone found in soil (Williamson *et al*., 2011). Bacteria belonging to this last phylum are barely studied and mostly uncultured nitrite-oxidizing bacteria, which are, according to molecular data, among the most diverse and widespread nitrifiers in natural ecosystems (Lücker *et al*., 2010). Finally, the *Gemmatimonadetes* (4.6%) and *Acidobacteria* (2.3%) phyla were both represented by sequences most related to uncultured bacterial clones commonly detected in soil samples.

The domain *Archaea* was detected on salt efflorescences in sample W2 (Table S2). All sequenced clones were closely related to different uncultured archaeons, in addition to the cultured genera *Halococcus* and *Halobacterium,* already recorded from other salt-attacked monuments (Piñar *et al*., 2009; Ettenauer *et al*., 2010). This study confirms results of previous works (Saiz-Jimenez & Laiz, 2000; Piñar *et al*., 2001, 2009; Imperi *et al*., 2007; Ettenauer *et al*., 2010), where the cohabitation of *Bacteria* and *Archaea* in salt-attacked monuments was observed. This can be due to the local high variations in salinity and pH present on these deteriorated environments.

In summary, the microbiota inhabiting the wall samples was determined by the environmental conditions found in the Catacombs, mainly by the crystallization of salts on the wall surfaces and this explains the dominant occurrence of halotolerant and halophilic species of the domains *Bacteria* and *Archaea*. The halophilic species of the *Gammaproteobacteria* (such as *Idiomarina*, *Salinisphaera,* and *Halomonas*), but also species of the phyla *Bacteroidetes* and *Actinobacteria* (such as *Rubrobacter*) as well as the haloarchaea detected on the walls of the Catacombs may be responsible for the extensive rosy discoloration observed. Their cell membranes contain carotenoid pigments such as β-carotene, α-bacterioruberin and derivatives, and salinixanthin, the latter discovered in halophilic species of the *Bacteroidetes*. These pigments primarily appear to protect the cells against photooxidative damage (Oren, 2009).

In addition, it is worth remarking on the presence of sequences related to pathogenic microorganisms and the human skin microbiome noted from wall samples, although in a lower proportion. The finding of these sequences may be related to the fact that the mummies are hanging directly on the walls, allowing a direct contact between wall and body materials and the cross-contamination of these materials by microorganisms.

### Samples from human remains

Six kinds of samples: M2+N2 (skin), P1 (muscle), M1+N3 (hair), C1+F6 (bones), C3 (stuffing material), and C4 (clothes) were analyzed by culture-independent techniques. All samples yielded pure DNA extracts with concentrations ranging between 112.13 and 383.55 ng DNA mg^−1^ of sample and amplifiable by PCR analysis. PCR analysis using eubacterial- and fungal-specific primers showed positive results for all samples collected from the mummies. Archaeal-specific primers yielded positive results for bone and stuffing samples, most probably due to the contamination of these materials by the salt detached from the walls, as archaeal DGGE-fingerprints derived from bone and stuffing samples showed to be almost identical to the fingerprint derived from wall material (see Fig. 4b, lanes 3, 7, and 9) and the sequenced clones showed the same phylogenetic affiliations (Table S2).

### Phylogenetic analyses of bacterial clones

**Figure 6:**
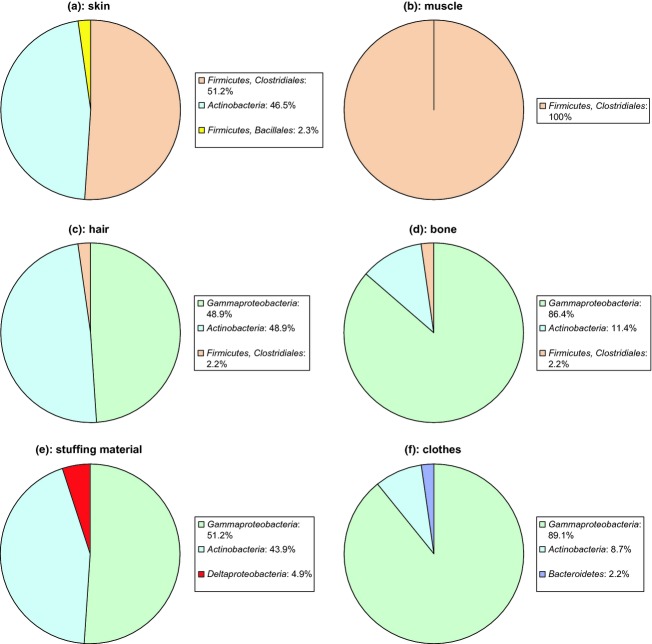
Diagram showing the relative abundance of the bacterial diversity on material obtained from and related with the mummies. (a) skin, (b) muscle, (c) hair, (d) bone, (e) stuffing material, and (f) clothes.

All clones wearing bacterial 16S rRNA gene fragments obtained from, and related with, materials of the mummies fell into four phylogenetic groups: *Proteobacteria* (*Gamma*- and *Delta*-classes), *Actinobacteria*, *Firmicutes* (order *Clostridiales* and *Bacillales*), and *Bacteroidetes* (Fig. 6 and Table S1b).

### Skin

In contrast to most of the other materials, no *Gammaproteobacteria* were found on skin (Fig. 6a). The order *Clostridiales* of the *Firmicutes* dominated on this material, being found in 51.2% of the screened clones. The detected sequences related to strains of *Clostridium botulinum* (48.8%) and to an uncultured bacterium clone detected on anaerobic digesters for animal waste treatment (2.3%). The order *Bacillales* of the *Firmicutes* accounted for 2.3% of the screened clones and showed to be affiliated with *Thermoactinomycetaceae* bacteria. The remaining clones (46.5%) affiliated with the *Actinobacteria* phylum, with sequences related to an uncultured *Pseudonocardiaceae* bacterium previously detected by molecular analysis on documents from a 16th century tomb. These documents were found on the pelvic region of a mummified body and were contaminated by body fluids and putrefaction (Jurado *et al*., 2010).

### Muscle

As observed for the skin, no *Gammaproteobacteria* were detected on muscle (Fig. 6b). On this material, all clones affiliated with members of the order *Clostridiales* of the *Firmicutes*, namely with *Sporanaerobacter* sp. C5BEL, a halotolerant bacterium isolated from sludge (Rezgui *et al*., 2012).

### Hair

On hair, 48.9% of the screened clones affiliated with members of the *Gammaproteobacteria* (Fig. 6c), namely with sequences of uncultured clones and halophilic species of the genera *Halomonas* (13.3%), *Chromohalobacter* (4.4%), and *Salinisphaera* (4.4%). In addition, sequences belonging to *Psychromonas arctica* (15.5%) possessing cold-active hydrolytic enzymes (Groudieva *et al*., 2004) and to the cultivated *Stenotrophomonas maltophilia* (8.9%), a well-known keratinolytic bacterium (Cao *et al*., 2009) were detected on this keratin-rich material. Finally, 2.2% of the clones showed to be related to luminous and nonluminous species of the genus *Photobacterium* (Ast & Dunlap, 2005). The phylum *Actinobacteria* showed to be as abundant as the *Gammaproteobateria* (48.9% of the screened clones), being all sequences related to uncultured and cultured species of the genus *Rubrobacter* (Imperi *et al*., 2007; Laiz *et al*., 2009; Jurado *et al*., 2012). Species of this genus have been frequently detected in connection with the rosy discoloration and formation of efflorescences on mural paintings and building materials. These sequences were also detected on the walls of the Catacombs, and therefore, the presence of these species on hair may be a contamination of this material by the salt detached from the walls. Finally, 2.2% of the screened clones found on hair showed to be affiliated with members of the *Firmicutes*, of the order *Clostridiales*. These sequences showed the highest score similarity with uncultured bacterial clones associated with the human skin microbiome (Grice *et al*., 2009) and with disease flares in children with atopic dermatitis (Kong *et al*., 2012).

### Bones

On bones, the *Gammaproteobacteria* accounted for 86.4% of the screened clones (Fig. 6d), with sequences related to halophilic species of the genera *Chromohalobacter* (9.1%), *Halomonas* (4.5%), and *Salinisphaera* (4.5%). However, in addition, sequences related to an uncultured clone detected on activated sludge and to cultivated *Luteibacter sp*. dominated on this material (68.2%). The *Actinobacteria* phylum accounted for the 11.4% of the screened clones. Some of these sequences (6.8%) were related to an uncultured bacterium clone found on human skin (Grice *et al*., 2009), as well as to cultivated species of the genus *Pseudonocardia*. The rest of the sequences (both accounting for 2.3% of the screened clones) were related to species of the genus *Streptomyces* and *Mycobacterium*, the latter being recovered from clinical specimens (Masaki *et al*., 2006). Finally, 2.3% of the screened clones found on bones showed to be affiliated with members of the *Firmicutes* of the order *Clostridiales*, namely with *Clostridium tetani,* the causative agent of tetanus disease (Brüggeman *et al*., 2003).

### Stuffing material

Halotolerant and halophilic species of the *Gammaproteobacteria* dominated on stuffing material appearing on 51.2% of the screened clones, and sequences showed to be related to the genera *Salinisphaera* (39%) and *Chromohalobacter* (12.2%) (Fig. 6e). In addition, 4.9% of the screened clones affiliated with members of the *Deltaproteobacteria*. This group of microorganisms was detected solely on the stuffing material, being the sequences related to an uncultured bacterium clone found on cellulosic waste containing known cellulose-degrading microorganisms (Field *et al*., 2010). The *Actinobacteria* detected on this material (accounting for the 43.9% of the screened clones) showed to be affiliated with species of *Arthrobacter*, such as *A. pigmenti* (9.8%), which was previously isolated from deteriorated mural paintings showing rosy discoloration (Heyrman *et al*., 2005); with species of the genus *Brachybacterium*, such as *B. fresconis* and *B. sacelli* (4.8%), both halotolerant and also detected on rosy discolored medieval wall paintings (Heyrman *et al*., 2002) and with *B. zhongshanense* (2.4%), a cellulose-decomposing bacterium (Zhang *et al*., 2007). In addition, 7.3% of the screened clones affiliated with species of cultivated *Cellulomonas* (Brito *et al*., 2006) and 4.8% affiliated with *Kocuria* spp. The last 19.4% of the screened clones showed the highest score similarity with an uncultured bacterium clone associated with disease flares in children with atopic dermatitis (Kong *et al*., 2012). It is important to remark that the stuffing material collected showed an intriguing rosy discoloration phenomenon. As mentioned for wall samples, the halophilic species of the *Gammaproteobacteria* and *Archaea* detected in this sample may be responsible for this discoloration, but in addition, the species of *Kocuria* and *Arthrobacter* detected on the stuffing material could increase this discoloration, as many species of these two genera are red-pigmented.

### Clothes

As observed on the stuffing material, halotolerant and halophilic species of the *Gammaproteobacteria* dominated as well on clothes (89.1% of screened clones), as sequences related to cultivated species of the genera *Halomonas* (71.7%), *Chromohalobacter* (15.2%), and *Salinisphaera* (2.2%) (Fig. 6f). The *Actinobacteria* accounted for the 8.7% of screened clones, with sequences related to the genera *Jiangella* (4.3%), *Pseudonocardia* (2.2%), and *Streptomonospora* (2.2%). These genera have been previously detected on mold-colonized water-damaged building materials (Schäfer *et al*., 2010) and caves (Lee, 2008; Hodges *et al*., 2012). In addition, 2.2% of the screened clones affiliated with the *Bacteroidetes* phylum, namely with species of the genus *Alifodinibius*, inhabiting halophilic environments.

In summary, sequence analyses evidenced a strong contamination of the mummies with halophilic microorganisms, deposited on their surfaces through detachment from the walls. However, besides this contamination, results showed that there was a specific colonization of materials by specialized microorganisms. The sequences detected on cellulosic compounds, such as clothes and stuffing materials, showed to be related to microorganisms that are known to possess cellulolytic activities. Those on keratin- and collagen-rich materials showed sequences related to microorganisms possessing proteolytic and keratinolytic activities. The only one material not contaminated by halophilic microorganisms was skin, probably due to the fact that the analyzed samples were collected from two mummies lying down within coffins and therefore protected from the salt contamination. This material proved to be entirely colonized by members of specific taxa known to harbor bacteria able to produce deterioration of this material.

### Phylogenetic analyses of fungi

Contrary to what was observed on the wall materials, the surface of many mummies – including the heads, clothes, and stuffing material – was heavily contaminated with molds (Fig. 1c). There was superficial growth of fungi, but also a deep infection of materials. Cultivation analyses were performed using contact plates to isolate the fungi proliferating on the surface of the mummies. Thirty-three different fungal strains were isolated from the surface and inside the mummy materials, some were common airborne fungi and some originating from the material used as stuffing in the clothing of the mummies.

The most prominent airborne fungal contaminations detected were: *Penicillium brevicompactum*, *P. chrysogenum*, *P. expansum,* and species of the genus *Aspergillus*. Fungal genera more related to the mummy materials were: *Botryotinia*, *Giberella*, *Didymella*, *Fusarium*, *Verticillium*, *Tritirachium*, *Coprinus,* and *Coniosporium*.

**Figure 7:**
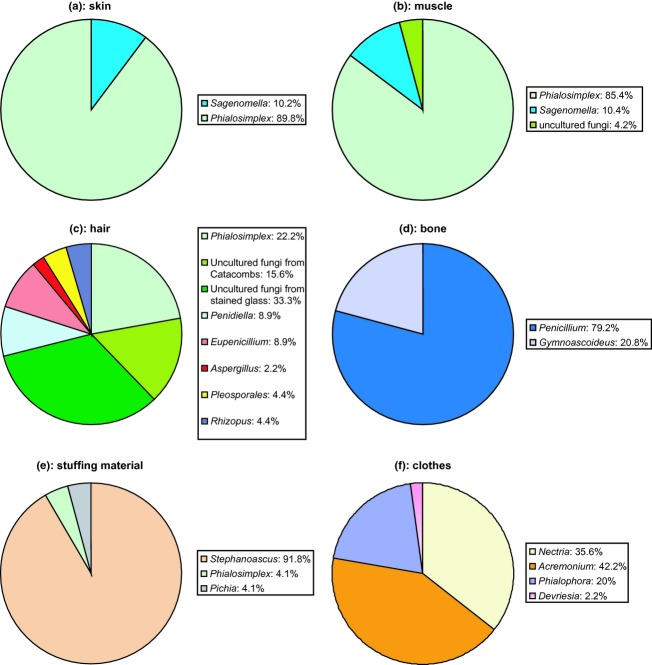
Diagram showing the relative abundance of the fungal diversity on material obtained from and related with the mummies. Legend as for Fig. 6.

Molecular analyses revealed that among fungi, sequences related to pathogenic species of the genus *Phialosimplex,* an ascomycete of the family *Trichocomaceae*, dominated on samples derived from the mummies, especially on skin (89.8% of the screened clones), muscle (85.4%), and hair (22.2%) – all of which are keratin- and collagen-rich materials – and in a lower proportion were also detected on the stuffing material (2%) (Fig. 7 and Table S3). Species of this genus have been associated with human and animal infections (Gené *et al*., 2003; Sigler *et al*., 2010), and more recently associated with the biodeterioration of collagenous materials, such as ancient parchments (Piñar *et al*., 2011). In addition, sequences related to pathogenic species of the genus *Sagenomella,* also members of the family *Trichocomaceae*, were detected on skin (10.2% of screened clones) and muscle (10.4%) (Fig. 7a and b). In the first material, these clones also showed high scores of similarity to an ascomycota sp. SAB-A1B-T isolated from textile materials inside a crypt (Pangallo *et al*., 2013). *Sagenomella* species have been described as the causative agents of fungal keratitis (Hsieh *et al*., 2009). The remaining clones screened from the muscle sample affiliated with uncultured fungal clones detected on continental and marine air (2.1%) and on dust from moisture-damaged buildings (2.1%) (Fig. 7b).

On hair (Fig. 7c), besides the *Philaosimplex* spp. (accounting for 22.2% of the screened clones), 15.6% of clones affiliated (97% similarity) with an uncultured clone detected during the archaeological excavation of Catacombs dated to the Xizhou Dynasty, China (Wu, F., Su, B., He, D., Chen, G. and Wang, W., unpublished). However, the closest cultivated phylogenetic relative of these clones was also *Phialosimplex* sp. (but with a 93% similarity). 33.3% of clones showed the highest score similarity to an uncultured fungal clone detected on decayed glass in Catalonian churches (Piñar *et al*., 2013). 8.9% of the screened clones were affiliated with species of the genus *Penidiella*, namely *P. venezuelensis*. This species has been isolated from men with *tinea nigra*, a superficial fungal infection that causes dark brown to black painless patches on the palms of the hands and the soles of the feet (Crous *et al*., 2007). In addition, species of the genera *Eupenicillium* (8.9%) and *Aspergillus* (2.2%), which are saprotrophic fungi (Daynes *et al*., 2012) were detected. The remaining clones were related to unidentified fungal species and cultivated species of the order *Pleosporales* (4.4%), detected in association with roots of halophytic plant species (Maciá-Vicente *et al*., 2012) and to *Rhizopus oryzae* (4.4%), a human pathogenic agent of mucormycosis (an invasive opportunistic infection caused by fungi belonging to the order *Mucorales*).

Surprisingly, on bone (Fig. 7d), sequences related to *Penicillium radicum* showed to be dominant and unique (79.1% of the screened clones). *P. radicum* is a phosphate-solubilizing fungus used in agriculture for promoting plant growth (Whitelaw *et al*., 1999). However, there is a more recent study reporting on a disseminated *P. radicum* infection in a dog (De Vos *et al*., 2009). The phosphate solubilization capability of this fungus could be involved in the solubilization of the phosphorus contained in bones. Hydroxyapatite (tricalcium phosphate) is the dominant mineral of bone, with smaller quantities of intermediate calcium phosphates and some calcium carbonate present. The rest of the screened clones of this sample (20.8%) affiliated with sequences related to species of the *Gymnoascoideus* genus.

On stuffing material (Fig. 7e), the sequences that showed to be most related to *Phialosimplex* spp. (4.1%) affiliated as well with uncultured clones detected in an underground archaeological excavation site (Wu, F., Su, B., He, D., Chen, G. and Wang, W., unpublished) and with an ascomycota isolated from textile materials inside a crypt (Pangallo *et al*., 2013). The other 95.9% screened clones proved to be related to yeast sequences, and 91.8% of them affiliated with species of the genus *Stephanoascus*, namely *Stephanoascus ciferrii*. This species is a teleomorph of *Candida ciferrii*. *Candida* species are mainly associated with plants, with rotting vegetation, with insects feeding on plants, or with food. In line with this, 71% of *Candida* species utilize xylose (wood degradation) and 57% of species use cellobiose (cellulose degradation) (Schauer & Hanschke, 1999). These enzymatic activities may be involved in the degradation of the two main materials used for stuffing the mummies: tow and straw (Piombino-Mascali *et al*., 2011). However, some studies have reported *C. ciferrii* (teleom. *Stephanoascus ciferrii*) to cause invasive fungal infections in humans (Gunsilius *et al*., 2001; Agin *et al*., 2011). The remaining 4.1% of the detected yeast sequences affiliated with an uncultured clone related with the genus *Pichia*, detected in agarwood.

On clothes (Fig. 7f), cultivated species of the genus *Acremonium* dominated (42.2%), but some of these sequences were also affiliated with uncultured clones detected in archaeological excavations and dust from moisture-damaged buildings. Members of this genus are well-known for their cellulolytic activities. *Nectria* spp. accounted for 35.5% of the screened clones. They are most often encountered as saprophytes on decaying wood, but some species can also occur as parasites of trees. Finally, species of the genus *Phialophora* (20%), also known for their cellulolytic activities, and of the genus *Devriesia* (2.2%) were detected.

As already observed for *Bacteria*, the analyses of the fungal communities revealed a specific colonization of different types of materials by specialized fungi. Sequences most related to fungi with known cellulolytic activities were found on clothes and stuffing material, whereas sequences related to fungi showing proteolytic and keratinolytic activities were detected on skin, muscle, and hair. Finally, on bone material, most of the detected sequences affiliated with a specialized fungus able to solubilize phosphate.

## Conclusions

This study provides an initial insight into the curatorial problems concerning the human remains located in the Capuchin Catacombs of Palermo. The sampling campaign performed at the Catacombs and the strategy used for the analysis of samples, combining conventional cultivation and molecular techniques, contributed to the success of this investigation.

The results of this research have suggested the following conclusions. Firstly, the indoor air quality showed a very high concentration of fungal spores not conducive to the conservation of the human remains. Furthermore, levels significant enough to pose a potential health risk to visitors were demonstrated. Secondly, the environmental conditions at the Catacombs have allowed the formation of salt deposits all over the walls, which offer a special habitat for very specialized halophilic microorganisms. These halophilic microorganisms are responsible for the extensive rosy discoloration observed on the Catacombs walls. Salt cracks are detaching and contaminating the surfaces of other mummified remains, including their clothes, hair, bones, and stuffing material, allowing the settlement of halophilic microorganisms on these materials. Finally, besides the superficial salt contamination, sequences most related to highly specialized *Bacteria* and fungi taxa, whose members are well-known for their proteolytic and keratinolytic activities, were detected on and inside the mummy materials, such as skin, muscle, and hair. On the other hand, sequences related to cellulolytic microorganisms were found on cellulosic compounds such as clothes and stuffing material. The high score of similarities observed between our sequences and microorganisms possessing specialized enzymatic activities suggest a specific colonization of the investigated materials as well as a potential relation with their deterioration.

Simple measurements such as an optimization of ventilation, and the removal of dust and detached salts by cleaning, would significantly improve the preservation of the mummies. The application of further disinfectant treatments to combat mold proliferation will have to be decided by restorers.

## Statement

The human mummies held in the Capuchin Catacombs of Palermo showed different biodegradation phenomena; hence, they were investigated by means of microbiological and molecular techniques; halophilic/halotolerant microorganisms as well as a specialized microbiota belonging to taxa well-known for their cellulolytic and proteolytic activities were identified; and a high concentration of airborne fungal spores was suggestive of a potential health risk for visitors.

## Supporting Information

Table S1a Phylogenetic affiliations of the partial 16S rRNA coding sequences obtained from wall materials collected in the Capuchin Catacombs, Palermo.

Table S1b Phylogenetic affiliations of the partial 16S rRNA coding sequences obtained from human remains held in the Capuchin Catacombs, Palermo.

Table S2 Phylogenetic affiliations of the archaeal partial 16S rRNA coding sequences detected in the Capuchin Catacombs, Palermo.

Table S3 Phylogenetic affiliations of the fungal ITS coding sequences obtained from human remains held in the Capuchin Catacombs, Palermo.
