# Supplementary material for: Microbial survey of the mummies from the Capuchin Catacombs of Palermo, Italy: biodeterioration risk and contamination of the indoor air
Source: FEMS Microbiol Ecol. 2013 Jul 9;86(2):341–56. doi: 10.1111/1574-6941.12165 (PMC3916889; doi:10.1111/1574-6941.12165)
Supplement: Table S1a — Phylogenetic affiliations of the partial 16S rRNA coding sequences obtained from wall materials collected in the Capuchin Catacombs, Palermo. [file fem0086-0341-sd1.docx]

**Table S1a.** Phylogenetic affiliations of the partial 16S rRNA coding sequences obtained from wall materials collected in the Capuchin Catacombs, Palermo.

| **Phylum** | **Clone (%)** | **Selected clone** | **Length**  [bp] | Closest identified phylogenetic relatives [EMBL accession numbers] | **Simil.**  **(%)** | **Accession**  **number** |
| --- | --- | --- | --- | --- | --- | --- |
| **Sample W1: rosy discoloration** | | | | | | |
| *Proteobacteria*  Gammaproteobact.  (39.5%) | 11.6% | B6-K16 | [627] | *Salinisphaera* spp. 16S ribosomal RNA gene, partial sequence [AB735546, EU143343, JN020587]. | 94 | KC535171 |
|  | 2.3% | B6-K44 | [645] | *Salinisphaera* spp. 16S ribosomal RNA gene, partial sequence [AB735546, EU143343, JN020587]. | 98 | KC535184 |
|  | 2.3% | B6-K25 | [644] | Uncultured gamma proteobacterium clone HG-J01225 16S ribosomal RNA gene, partial sequence [JN408970] from rhizosphere soil.  *Halomonas* spp. 16S ribosomal RNA, partial sequence [NR_044350, HE964771, NR_025486], moderately halophilic bacteria. | 99  98 | KC535175 |
|  | 4.6% | B6-K27 | [644] | Uncultured bacterial clones 16S ribosomal RNA gene, partial sequence [EU735714, EU735712] from groundwater aquifer.  *Halomonas* spp. 16S ribosomal RNA gene, partial sequence [EU447169, EU447166], haloalkaliphilic Gammaproteobacteria from hypersaline soda lakes. | 98  98 | KC535177 |
|  | 7% | B6-K29 | [644] | Uncultured bacterial clones 16S ribosomal RNA gene, partial sequence [EU735714, EU735712] from groundwater aquifer.  *Halomonas* spp. 16S ribosomal RNA gene, partial sequence [EU447169, EU447166], haloalkaliphilic Gammaproteobacteria from hypersaline soda lakes. | 98  98 | KC535178 |
|  | 2.3% | B6-K3 | [645] | Uncultured bacterial clones [KC408579, HQ792347, HQ792248].  *Acinetobacter* spp. 16S ribosomal RNA gene, partial sequence [JX022748, EU916711, EU916708, JF915343]. | 99  99 | KC535166 |
|  | 2.3% | B6-K12 | [645] | Uncultured bacterial clones 16S ribosomal RNA gene, partial sequence [JF206792, HM344173, HM320901] associated with disease flares and treatment in children with atopic dermatitis.  *Acinetobacter* spp. 16S ribosomal RNA gene, partial sequence [KC790286, KC790277, KC758136]. | 100  99 | KC535168 |
|  | 2.3% | B6-K21 | [645] | *Acinetobacter* spp. 16S ribosomal RNA gene, partial sequence [KC422447, JN228308, JN187418]. | 100 | KC535173 |
|  | 2.3% | B6-K32 | [645] | Uncultured bacterium clone ncd2607b06c1 16S ribosomal RNA gene, partial sequence [JF228130] associated with disease flares and treatment in children with atopic dermatitis.  *Acinetobacter* spp. [KC753351, JQ765576, JQ359095, HM584279]. | 100  99 | KC535181 |
|  | 2.3% | B6-K5 | [645] | Uncultured bacterial clones 16S ribosomal RNA gene, partial sequence [HQ115539, HM238165, GU205718].  *Legionella* spp. 16S ribosomal RNA gene, partial sequence [JN381004, NR_036804]. | 97  96 | KC535167 |
| Alphaproteobact.  (2.3%) | 2.3% | B6-K40 | [618] | Uncultured bacterium clone 8619_plate2d09 16S ribosomal RNA gene, partial sequence [HM356924] from ancient algal mats.  *Devosia chinhatensis* strain IPL18 16S ribosomal RNA, partial sequence [NR_044214] isolated from a hexachlorocyclohexane (HCH) dump site. | 99  97 | KC535183 |
| Betaproteobact.  (2.3%) | 2.3% | B6-K15 | [646] | *Cupriavidus metallidurans* partial 16S rRNA gene [JQ046372, NR_074704]. | 99 | KC535170 |
| *Actinobacteria*  (21%) | 11.6% | B6-K30 | [630] | *Corynebacterium kroppenstedtii* DSM 44385 complete genome [CP001620, NR_074408] recovered from human sputum, and isolate 00-0244 16S ribosomal RNA gene, partial sequence [AF537598] recovered from human clinical material in Canada. | 100 | KC535179 |
|  | 4.6% | B6-K22 | [631] | Uncultured bacterial clones 16S ribosomal RNA gene, partial sequence [JF095491, JF089698, JF095549] associated with disease flares and treatment in children with atopic dermatitis.  *Ruania albidiflava* strain AS 4.3142 16S ribosomal RNA, partial sequence [NR_043736]. | 99  97 | KC535174 |
|  | 2.3% | B6-K26 | [632] | Uncultured Frankineae bacterium clone LWM1-63 16S ribosomal RNA gene, partial sequence [HQ674869] on weathered feldspar mineral.  *Frankia* spp. 16S ribosomal RNA, complete sequence [NR_074514, JN685211, JN698222]. | 94  94 | KC535176 |
|  | 2.3% | B6-K31 | [644] | Uncultured bacterium clone FFCH3290 16S ribosomal RNA gene, partial sequence [EU133067], member of the soil biosphere.  Bacterium Ellin6526 16S ribosomal RNA gene, partial sequence [HM748674], very slow-growing and mini-colony-forming soil Rubrobacteridae bacteria. | 99  96 | KC535180 |
| *Firmicutes*  Bacillales  (21%) | 21% | B6-K14 | [644] | *Paenibacillus* spp. 16S ribosomal RNA gene, partial sequence [JQ977352, KC753337, KC355299, KC462533]. | 100 | KC535169 |
| *Firmicutes*  Clostridiales  (11.6%) | 11.6% | B6-K19 | [619] | *Clostridium botulinum* strains, 16S ribosomal RNA gene, partial sequence [KC331205, JN617090, JN617056, CP001078]. | 100 | KC535172 |
| *Bacteroidetes*  (2.3% | 2.3% | B6-K38 | [636] | *Bacteroidetes* bacterium YIM D15 16S ribosomal RNA gene, partial sequence [JQ923475], *Alifodinibius roseus* gen. nov., sp. nov., and *Alifodinibius sediminis* sp. nov., isolated from salt mine sample. | 98 | KC535182 |
| **Sample W2: salt efflorescence** | | | | | | |
| *Proteobacteria*  Gammaproteobact.  (34.8%) | 2.2% | B7-K43 | [645] | *Salinisphaera* spp. 16S ribosomal RNA gene, partial sequence [AB735546, EU143343, JN020587]. | 98 | KC535232 |
|  | 2.2% | B7-K4 | [644] | Uncultured bacterial clones 16S ribosomal RNA gene, partial sequence [EF157249, EF157230] in natural asphalts.  *Halomonas muralis* partial 16S rRNA gene [AJ320531, AJ320532, NR_025486] isolated from microbial biofilms colonizing the walls and murals of the Catherine Chapel (Herberstein Castle, Austria). | 99  99 | KC535221 |
|  | 2.2% | B7-K7 | [637] | *Halomonas muralis* partial 16S rRNA gene [AJ320531, AJ320532, NR_025486] isolated from microbial biofilms colonizing the walls and murals of the Catherine Chapel (Herberstein Castle, Austria). | 99 | KC535224 |
|  | 23.9% | B7-K8 | [635] | *Idiomarina* spp. partial 16S rRNA gene, strain RHS-str.1044JA [KC753341, KC583216, HE586864]. | 96 | KC535225 |
|  | 4.3% | B7-K5 | [645] | Uncultured bacterium clone J1-BUN 16S ribosomal RNA gene, partial sequence [EF174281] from activated sludge.  *Luteibacter rhizovicinus* gene for 16S rRNA, partial sequence [AB627008, NR_042197]. | 97  97 | KC535222 |
| *Actinobacteria*  (32.6%) | 2.2% | B7-K1 | [644] | Uncultured bacterium clone nbw120g09c1 16S ribosomal RNA gene, partial sequence [GQ008796] from the human skin microbiome.  *Rubrobacter bracarensis* isolates [HE672088, HE672086, HE672087], novel members of the Rubrobacteraceae family isolated from a deteriorated monument. | 99  99 | KC535218 |
|  | 6.5% | B7-K6 | [643] | *Rubrobacter bracarensis* isolates [HE672088, HE672086, HE672087], novel members of the Rubrobacteraceae family isolated from a deteriorated monument. | 99 | KC535223 |
|  | 19.6% | B7-K27 | [644] | Uncultured bacterium clone nbw120g09c1 16S ribosomal RNA gene, partial sequence [GQ008796,] from the human skin microbiome.  *Rubrobacter bracarensis* isolates [EU512989, HE672088, HE672086, HE672087], novel members of the Rubrobacteraceae family isolated from a deteriorated monument. | 99  99 | KC535229 |
|  | 4.3% | B7-K34 | [641] | Uncultured bacterium clone P7-WPA73-90 16S ribosomal RNA gene, partial sequence [GU574026], Actinobacteria from mould-colonized water-damaged building material. | 99 | KC535230 |
| *Firmicutes*  Bacillales  (28.3%) | 2.2% | B7-K2 | [646] | *Sediminibacillus albus* strain NHBX5 16S ribosomal RNA, partial sequence [NR_044031] isolated from a hypersaline lake. | 99 | KC535219 |
|  | 2.2% | B7-K26 | [646] | *Sediminibacillus albus* strain NHBX5 16S ribosomal RNA, partial sequence [NR_044031] isolated from a hypersaline lake. | 99 | KC535228 |
|  | 10.9% | B7-K40 | [646] | *Sediminibacillus albus* strain NHBX5 16S ribosomal RNA, partial sequence [NR_044031] isolated from a hypersaline lake. | 99 | KC535231 |
|  | 6.5% | B7-K19 | [642] | Uncultured bacterium clone CS1-1 16S ribosomal RNA gene, partial sequence [EU620444], facultative alkalitolerant and halotolerant endospore former in soil.  *Bacillus mannanilyticus* partial 16S rRNA gene, strain IB-OR17-B1 [HE663240], alkaliphilic bacterial strain producing extracellular antifungal compounds and chitinase and strain AM-001 16S ribosomal RNA, complete sequence [NR_040851], alkaliphilic strains. | 99  99 | KC535226 |
|  | 6.5% | B7-K21 | [643] | Uncultured bacterium clone AKIW584 16S ribosomal RNA gene, partial sequence [DQ129335] from urban aerosols.  *Thermoactinomyces sacchari* 16S ribosomal RNA gene, partial sequence [AF089890], thermophilic Actinomycete-like bacteria involved in hypersensitivity pneumonitis. | 99  99 | KC535227 |
| *Bacteroidetes*  (4.3%) | 4.3% | B7-K3 | [643] | Uncultured bacterium clones 16S ribosomal RNA gene, partial sequence [GQ262865, GQ262972], influence of cellulosic waste on the bacterial community structure at a simulated low-level-radioactive-waste site.  *Balneola* spp. partial 16S rRNA gene [AM990892, NR_044367, NR_042991] in Mediterranean ecosystem. | 99  90 | KC535220 |
| **Sample PS: purple stains** | | | | | | |
| *Proteobacteria*  Gammaproteobact. (13.6%) | 9.1% | B8-K26 | [644] | *Cellvibrio fibrivorans* strain R-4079 16S ribosomal RNA, partial sequence [NR_025420], cellulolytic bacteria involved in the degradation of natural cellulosic fibres. | 99 | KC535158 |
|  | 2.3% | B8-K32 | [636] | Uncultured bacterial clones 16S ribosomal RNA gene, partial sequence [GQ262966, GQ262964], influence of cellulosic waste, and [JN801110, JN801104] of the lava tubes in Pico island, Azores. | 98 | KC535160 |
|  | 2.3% | B8-K10 | [637] | *Moraxella* spp. 16S ribosomal RNA gene, partial sequence [KC810841, KC693705, KC456542]. | 100 | KC535154 |
| Alphaproteobact.  (9.1%) | 2.3% | B8-K35 | [612] | Uncultured bacterial clones 16S ribosomal RNA gene, partial sequence [FN429563, HM126845, HQ697510] with biodegradation capabilities.  *Hyphomicrobium hollandicum* strain IFAM KB-677 16S ribosomal RNA, partial sequence [NR_026428]. | 99  97 | KC535162 |
|  | 4.5% | B8-K36 | [609] | Uncultured alpha proteobacteria clones 16S ribosomal RNA gene, partial sequence [HM111812, HM111662] associated with honey bees and bumble bees.  *Hyphomicrobium* spp. chromosome, complete genome [NR_074190, FQ859181]. | 98  98 | KC535163 |
|  | 2.3% | B8-K16 | [618] | Uncultured bacterium clone BF_A11-17 16S ribosomal RNA gene, partial sequence [KC238348] from seawater.  Parvularculaceae bacterium P33 16S ribosomal RNA gene, partial sequence [EU851414] isolated from deep sea water. | 99  99 | KC535156 |
| *Actinobacteria*  (27.3%) | 6.8% | B8-K1 | [644] | *Rubrobacter bracarensis* isolates [HE672088, HE672086, HE672087], novel members of the Rubrobacteraceae family isolated from a deteriorated monument. | 99 | KC535147 |
|  | 2.3% | B8-K12 | [644] | *Rubrobacter* spp. 16S ribosomal RNA gene, partial sequence [EU512989, HE672086] from biodeteriorated monuments. | 99 | KC535155 |
|  | 2.3% | B8-K17 | [644] | Uncultured bacterium clone LL141-8C18 16S ribosomal RNA gene, partial sequence [FJ675406].  Uncultured Rubrobacteridae bacterial partial 16S rRNA gene clones from hydrocarbon-contaminated soil [AM936423, AM935655]. | 99  99 | KC535157 |
|  | 9.1% | B8-K6 | [636] | *Nocardioides hwasunensis* strains ribosomal RNA, partial sequence [NR_042546, AM295257]. | 100 | KC535152 |
|  | 2.3% | B8-K44 | [626] | *Nocardioides* sp. Sco-B10 partial 16S rRNA gene, strain Sco-B10 [FN386732] isolated from volcanic ash. | 99 | KC535165 |
|  | 2.3% | B8-K7 | [626] | *Mycobacterium poriferae* strains 16S ribosomal RNA gene, partial sequence [JN627173, JN627174, JN627175, JN627176, JN627177] isolated from the East China Sea soft coral.  *Mycobacterium* spp. 6S ribosomal RNA gene, partial sequence [HM210419, HM210421, HM210422, HM210425] from marine sponges. | 99  99 | KC535153 |
|  | 2.3% | B8-K39 | [554] | *Pseudonocardia alaniniphila* strain 14613 16S ribosomal RNA, partial sequence [JN180176].  *Pseudonocardia petroleophila* strain IMSNU 22072 16S ribosomal RNA, complete sequence [NR_042005]. | 99  99 | KC535164 |
| *Bacteroidetes*  (13.6%) | 13.6% | B8-K5 | [351] | *Bacteroidetes* bacterium YIM D15 16S ribosomal RNA gene, partial sequence [JQ923475] *Alifodinibius roseus* gen. nov., sp. nov., and *Alifodinibius sediminis* sp. nov., isolated from salt mine sample. | 99 | KC535151 |
| *Chloroflexi*  (13.6%) | 11.3% | B8-K4 | [619] | Uncultured bacterium clones 16S ribosomal RNA gene, partial sequence [KC683094] from rivers impacted by mining.  *Dehalococcoides* spp., complete genome [CP001924, CP000688]. | 99  87 | KC535150 |
|  | 2.3% | B8-K31 | [622] | Uncultured bacterium clones 16S ribosomal RNA gene, partial sequences [JQ978845, GQ425394, FJ675508].  *Sphaerobacter thermophilus* DSM 20745 strain DSM 20745 16S ribosomal RNA, complete sequence [NR_042118]. | 97  87 | KC535159 |
| *Nitrospirae*  (15.9%) | 15.9% | B8-K2 | [631] | Uncultured bacterium 1112855455225 16S ribosomal RNA gene, partial sequence [HQ118336], prokaryotic DNA from soils.  Candidatus *Nitrospira defluvii* chromosome, complete genome [NR_074700], nitrite-oxidizing bacteria. | 99  99 | KC535148 |
| *Gemmatimona-detes*  4.6%) | 4.6% | B8-K34 | [609] | Uncultured Gemmatimonadetes bacterium clone HG-B01241 16S ribosomal RNA gene, partial sequence [JN409149] detected in rhizosphere soil.  *Gemmatimonas aurantiaca* T-27 strain T-27 (= NBRC 100505) 16S ribosomal RNA, complete sequence [NR_074708]. | 97  90 | KC535161 |
| *Acidobacteria*  (2.3%) | 2.3% | B8-K3 | [608] | Uncultured Acidobacteria bacterial clones 16S ribosomal RNA gene, partial sequence [HQ597771, HQ729842] from soils.  Acidobacteria bacterium KBS 96 16S ribosomal RNA gene, parcial sequence [FJ870384]. | 99  90 | KC535149 |
