# Supplementary material for: Microbial survey of the mummies from the Capuchin Catacombs of Palermo, Italy: biodeterioration risk and contamination of the indoor air
Source: FEMS Microbiol Ecol. 2013 Jul 9;86(2):341–56. doi: 10.1111/1574-6941.12165 (PMC3916889; doi:10.1111/1574-6941.12165)
Supplement: Table S1b — Phylogenetic affiliations of the partial 16S rRNA coding sequences obtained from human remains held in the Capuchin Catacombs, Palermo. [file fem0086-0341-sd2.docx]

**Table S1b.** Phylogenetic affiliations of the partial 16S rRNA coding sequences obtained from human remains held in the Capuchin Catacombs, Palermo.

| **Phylum** | **Clone**  **(%)** | **Selectedclone** | **Length**  [bp] | Closest identified phylogenetic relatives [EMBL accession numbers] | **Simil.**  **%** | **Accession**  **number** |
| --- | --- | --- | --- | --- | --- | --- |
| **Sample**  **M2+N2: skin** | | | | | | |
| *Firmicutes*  Clostridiales  (51.2%) | 46.6% | B5-K1 | [619] | *Clostridium botulinum* strains 16S ribosomal RNA gene, partial sequence [KC331205, KC331190] [JN617090, JN617091] [CP001078, CP001056] [EF030540, EF030541] [X68170, X68171, X68173]. | 100 | KC535233 |
|  | 2.3% | B5-K11 | [619] | *Clostridium botulinum* strains 16S ribosomal RNA gene, partial sequence [KC331205, KC331190] [JN617090, JN617091] [CP001078, CP001056] [EF030540, EF030541] [X68170, X68171, X68173]. | 99 | KC535236 |
|  | 2.3% | B5-K17 | [614] | Uncultured bacterium clone 02b06 16S ribosomal RNA gene, partial sequence [GQ133215] from anaerobic digesters for animal waste treatment.  *Tissierella* spp. 16S ribosomal RNA gene, partial sequence [GQ461815, GQ461817, GQ461819] isolated from human clinical samples. | 99  97 | KC535237 |
| *Firmicutes*  Bacillales  (2.3%) | 2.3% | B5-K23 | [644] | Thermoactinomycetaceae bacterium NariEX 16S ribosomal RNA gene, partial sequence [HQ383683] isolated from a salt lake.  Thermoactinomycetaceae bacteria 16S ribosomal RNA gene, partial sequence [HM032706, HM032707] isolated from human blood cultures. | 98  98 | KC535239 |
| *Actinobacteria*  (46.5%) | 34.9% | B5-K3 | [640] | Uncultured Pseudonocardiaceae bacterium partial 16S rRNA gene, clone IZ35.2_K11 [FN689586] from a microbiological study of bulls of indulgence of the 15^th^-16^th^ centuries.  *Saccharopolyspora* sp. TRM 45123 16S ribosomal RNA gene, partial sequence [KC295227]. | 99  96 | KC535234 |
|  | 6.9% | B5-K6 | [638] | Uncultured Pseudonocardiaceae bacterium partial 16S rRNA gene, clone IZ35.2_K11 [FN689586] from a microbiological study of bulls of indulgence of the 15^th^ -16^th^ centuries.  *Saccharopolyspora* sp. TRM 45123 16S ribosomal RNA gene, partial sequence [KC295227]. | 98  95 | KC535235 |
|  | 4.6% | B5-K22 | [638] | Uncultured Pseudonocardiaceae bacterium partial 16S rRNA gene, clone IZ35.2_K11 [FN689586] from a microbiological study of bulls of indulgence of the 15^th^-16^th^ centuries.  *Saccharopolyspora* sp. TRM 45123 16S ribosomal RNA gene, partial sequence [KC295227]. | 97  95 | KC535238 |
| **Sample P1: muscle** | | | | | | |
| *Firmicutes*  Clostridiales  (100%) | 90.3% | B1-K25 | [616] | *Sporanaerobacter* sp. C5BEL 16S ribosomal RNA gene, partial sequence [HQ534365], a halotolerant bacterium isolated from sludge. | 99 | KC535241 |
|  | 4.9% | B1-K15 | [616] | *Sporanaerobacter* sp. C5BEL 16S ribosomal RNA gene, partial sequence [HQ534365], a halotolerant bacterium isolated from sludge. | 98 | KC535240 |
|  | 2.4% | B1-K28 | [616] | *Sporanaerobacter* sp. C5BEL 16S ribosomal RNA gene, partial sequence [HQ534365], a halotolerant bacterium isolated from sludge. | 98 | KC535242 |
|  | 2.4% | B1-K32 | [616] | *Sporanaerobacter* sp. C5BEL 16S ribosomal RNA gene, partial sequence [HQ534365], a halotolerant bacterium isolated from sludge. | 98 | KC535243 |
| **Sample**  **M1+N3: hair** | | | | | | |
| *Proteobacteria*  Gammaproteobact.  (48.9%) | 8.9% | B4-K4 | [644] | Uncultured bacterial clones ribosomal RNA gene, partial sequence [EU735712, EU735714] from groundwater aquifer.  *Halomonas muralis* partial 16S rRNA gene [AJ320531, AJ320532, NR_025486] isolated from microbial biofilms colonizing the walls and murals of the Catherine Chapel (Herberstein Castle, Austria). | 99  98 | KC535187 |
|  | 2.2% | B4-K22 | [644] | Uncultured bacterium clones 16S ribosomal RNA gene, partial sequence [JN408970, JQ690675].  *Halomonas muralis* partial 16S rRNA gene [AJ320531, AJ320532, NR_025486] isolated from microbial biofilms colonizing the walls and murals of the Catherine Chapel (Herberstein Castle, Austria). | 99  98 | KC535194 |
|  | 2.2% | B4-K50 | [644] | Uncultured bacterial clones ribosomal RNA gene, partial sequence [EU735712, EU735714] from groundwater aquifer.  *Halomonas* spp. 16S ribosomal RNA gene, partial sequence [EU447166, EU447169], denitrification at extremely high salt and pH by haloalkaliphilic Gammaproteobacteria. | 99  98 | KC535201 |
|  | 4.4% | B4-K2 | [644] | *Chromohalobacter canadensis* strain RS93 16S ribosomal RNA gene, partial sequence [JQ638653] from potassium salt mine.  *Chromohalobacter beijerinckii* strain: NBRC 10304116S ribosomal RNA gene, partial sequence [AB681921].  *Chromohalobacter japonicus* strain 43 16S ribosomal RNA, partial sequence [NR_040965] from salty soils. | 99  99  99 | KC535186 |
|  | 2.2% | B4-K26 | [645] | *Salinisphaera* spp. 16S ribosomal RNA gene, partial sequence [AB735546, GU452539, EU143343, JN020587, HM137559], halophilic bacteria. | 98 | KC535195 |
|  | 2.2% | B4-K49 | [645] | *Salinisphaera* spp. 16S ribosomal RNA gene, partial sequence [AB735546, EU740414, EU740416, EU143343], halophilic bacteria. | 98 | KC535200 |
|  | 2.2% | B4-K7 | [640] | *Psychromonas arctica* strains 16S ribosomal RNA, complete sequence [JX173509, NR_028821], cold-active hydrolytic enzymes of culturable bacteria associated with Arctic sea ice, Spitzbergen. | 99 | KC535189 |
|  | 13.3% | B4-K8 | [644] | *Psychromonas arctica* strains 16S ribosomal RNA, complete sequence [JX173509, NR_028821], cold-active hydrolytic enzymes of culturable bacteria associated with Arctic sea ice, Spitzbergen. | 99 | KC535190 |
|  | 6.7% | B4-K9 | [637] | Uncultured bacterium clone BIGO1339 16S ribosomal RNA gene, partial sequence [HM558454], insect herbivore microbiome with high plant biomass-degrading capacity.  *Stenotrophomonas maltophilia* strains 16S ribosomal RNA gene [HF545327, KC252696, CP002986, HM584272, JF330158]. | 100  99 | KC535191 |
|  | 2.2% | B4-K12 | [645] | *Stenotrophomonas maltophilia* strains 16S ribosomal RNA gene [HF545327, KC252696, CP002986, HM584272, JF330158]. | 99 | KC535192 |
|  | 2.2% | B4-K47 | [644] | *Photobacterium iliopiscarium* strain NCIMB 13476 16S ribosomal RNA gene, partial sequence [AY849429].  *Photobacterium kishitanii* strain calba.5.9 16S ribosomal RNA gene, partial sequence [AY642170]. | 99  99 | KC535198 |
| *Actinobacteria*  (48.9%) | 35.5% | B4-K5 | [644] | Uncultured bacteria partial 16S rRNA gene clone [AM746681], bacterial etiology of rosy discoloration of ancient wall paintings.  *Rubrobacter* sp. 16S ribosomal RNA gene [EU512989] from biodeteriorated monuments. | 99  98 | KC535188 |
|  | 4.4% | B4-K1 | [644] | Uncultured bacteria partial 16S rRNA gene clone [AM746681], bacterial etiology of rosy discoloration of ancient wall paintings.  *Rubrobacter* sp. 16S ribosomal RNA gene [EU512989] from biodeteriorated monuments. | 98  98 | KC535185 |
|  | 4.4% | B4-K14 | [644] | Uncultured bacterial clones, 16S ribosomal RNA gene, partial sequence [GQ008796, GQ008744] from the human skin microbiome.  *Rubrobacter bracarensis* spp. partial 16S rRNA gene [HE672086, HE672087, HE672088], a novel member of the genus *Rubrobacter* isolated from a biodeteriorated monument. | 99  99 | KC535193 |
|  | 2.2% | B4-K33 | [639] | Uncultured bacteria partial 16S rRNA gene, clones [AM746681], bacterial etiology of rosy discoloration of ancient wall paintings.  *Rubrobacter* spp. 16S ribosomal RNA gene [EU512989] from biodeteriorated monuments. | 99  98 | KC535196 |
|  | 2.2% | B4-K48 | [644] | Uncultured bacteria partial 16S rRNA gene, clone [AM746681] bacterial etiology of rosy discoloration of ancient wall paintings.  *Rubrobacter* spp. 16S ribosomal RNA gene [EU512989] from biodeteriorated monuments. | 99  98 | KC535199 |
| *Firmicutes*  Clostridiales  (2.2%) | 2.2% | B4-K36 | [615] | Uncultured bacterium clone 16S ribosomal RNA gene, partial sequence [GQ026340] from the human skin microbiome.  Uncultured bacterial clones, 16S ribosomal RNA gene, partial sequence [JF178675, JF168303, JF104803, JF168916, JF108454] from the skin microbiome associated with disease flares and treatment in children with atopic dermatitis.  *Finegoldia magna* ATCC 29328 strain ATCC 29328 16S ribosomal RNA, complete sequence [NR_074677], an anaerobic opportunistic pathogen. | 100  100  99 | KC535197 |
| **Sample**  **C1+F6: bones** | | | | | | |
| *Proteobacteria*  Gammaproteobact.  (86.4%) | 4.5% | B3-K1 | [644] | *Chromohalobacter canadensis* strain RS93 16S ribosomal RNA gene, partial sequence [JQ638653] from potassium salt mine.  *Chromohalobacter beijerinckii* strain: NBRC 10304116S ribosomal RNA gene, partial sequence [AB681921].  *Chromohalobacter japonicus* strain 43 16S ribosomal RNA, partial sequence [NR_040965] from salty soils. | 100  100  100 | KC535244 |
|  | 2.3% | B3-K26 | [644] | *Chromohalobacter* sp. DS75-5 16S ribosomal RNA gene, partial sequence [JN196508, JN196509, JN196510, JN196511, JN196512] in hypersaline environments.  *Chromohalobacter nigrandesensis* strain Taxon 4 16S ribosomal RNA, partial sequence [NR_042011], a moderately halophilic, Gram-negative bacterium isolated from Lake Tebenquiche, Atacama Saltern, Chile. | 99  99 | KC535251 |
|  | 2.3% | B3-K37 | [644] | *Chromohalobacter canadensis* strain RS93 16S ribosomal RNA gene, partial sequence [JQ638653] from potassium salt mine.  *Chromohalobacter beijerinckii* strain: NBRC 10304116S ribosomal RNA gene, partial sequence [AB681921].  *Chromohalobacter japonicus* strain 43 16S ribosomal RNA, partial sequence [NR_040965] from salty soils. | 99  99  99 | KC535256 |
|  | 2.3% | B3-K10 | [645] | *Halomonas* spp. 16S ribosomal RNA gene, partial sequence. [JF710973, JF710974, JF710976, JF710977].  *Halomonas salina* strain CCMM B594 16S ribosomal RNA gene, partial sequence [JN208124], moderately halophilic and halotolerant bacteria. | 94  94 | KC535247 |
|  | 2.3% | B3-K22 | [644] | *Halomonas* spp*.* 16S ribosomal RNA gene, partial sequence [JQ716248, JQ716230]. | 99 | KC535250 |
|  | 4.5% | B3-K12 | [645] | *Salinisphaera japonica* YTM-1 gene for 16S ribosomal RNA, partial sequence [AB735546], a novel moderately halophilic bacterium isolated from the body surface of a deep-sea fish. | 98 | KC535248 |
|  | 59.1% | B3-K8 | [645] | Uncultured bacterium clone J1-BUN 16S ribosomal RNA gene, partial sequence [EF174281] from activated sludge.  *Luteibacter rhizovicinus* gene for 16S rRNA, partial sequence [AB627008]. | 97  96 | KC535246 |
|  | 6.8% | B3-K6 | [645] | Uncultured bacterium clone J1-BUN 16S ribosomal RNA gene, partial sequence [EF174281] from activated sludge.  *Luteibacter rhizovicinus* gene for 16S rRNA, partial sequence [AB627008]. | 97  97 | KC535245 |
|  | 2.3% | B3-K38 | [645] | Uncultured bacterium clone J1-BUN 16S ribosomal RNA gene, partial sequence [EF174281] from activated sludge.  *Luteibacter rhizovicinus* 16S ribosomal RNA, partial sequence [AB272380]. | 97  97 | KC535257 |
| *Actinobacteria*  (11.4%) | 2.3% | B3-K32 | [630] | Uncultured bacterium clone nbw113d01c1 16S ribosomal RNA gene, partial sequence [GQ008081], diversity of the human skin microbiome.  *Pseudonocardia* sp. strains 16S ribosomal RNA gene, partial sequence [GU318372, NR_042006]. | 99  99 | KC535253 |
|  | 4.5% | B3-K34 | [629] | Uncultured bacterium clone nbw113d01c1 16S ribosomal RNA gene, partial sequence [GQ008081], diversity of the human skin microbiome.  *Pseudonocardia* sp. strains 16S ribosomal RNA gene, partial sequence [GU318372, NR_042006]. | 97  96 | KC535254 |
|  | 2.3% | B3-K15 | [639] | Actinomycetales bacteria 16S ribosomal RNA gene, partial sequence [JQ924130, JQ924125] from marine sediment.  *Streptomyces vitaminophilus* strain NBRC 14294 16S ribosomal RNA, partial sequence [NR_041162]. | 95  95 | KC535249 |
|  | 2.3% | B3-K35 | [642] | *Mycobacterium longobardum* strain DSM 45394 16S ribosomal RNA gene, partial sequence [JN571166]. | 96 | KC535255 |
| *Firmicutes* Clostridiales  (2.2%) | 2.3% | B3-K30 | [618] | *Clostridium tetani* E88 strain Massachusetts 16S ribosomal RNA, complete sequence [NR_074498], the causative agent of tetanus disease.  *Clostridium tetani* strain NCTC 279 16S ribosomal RNA, partial sequence [NR_029260]. | 99  99 | KC535252 |
| **Sample C3 : stuffing material** | | | | | | |
| *Proteobacteria*  Gammaproteobact.  (51.2%) | 36.6% | B9-K7 | [434] | *Salinisphaera* spp. 16S ribosomal RNA gene, partial sequence [AB735546, EU143343], halophilic bacteria. | 98 | KC535282 |
|  | 2.4% | B9-K11 | [645] | *Salinisphaera* spp. 16S ribosomal RNA gene, partial sequence [GU452539, EU143343, JN020587, HM137559], halophilic bacteria. | 97 | KC535283 |
|  | 12.2% | B9-K26 | [591] | *Chromohalobacter canadensis* strain RS93 16S ribosomal RNA gene, partial sequence [JQ638653] from potassium salt mine.  *Chromohalobacter beijerinckii* strain: NBRC 10304116S ribosomal RNA gene, partial sequence [AB681921].  *Chromohalobacter japonicus* strain 43 16S ribosomal RNA, partial sequence [NR_040965] from salty soils. | 97  97  97 | KC535287 |
| Deltaproteobact.  (4.9%) | 4.9% | B9-K37 | [647] | Uncultured bacterium clone WC3_27 16S ribosomal RNA gene, partial sequence [GQ264098] from cellulosic waste.  *Desulfuromonas alkaliphilus* strain Z-0531 16S ribosomal RNA, partial sequence [NR_043709], alkaliphilic representative of the family Geobacteraceae, isolated from a soda lake. | 96  86 | KC535292 |
| *Actinobacteria*  (43.9%) | 9.8% | B9-K40 | [640] | *Arthrobacter* spp. 16S ribosomal RNA gene, partial sequence [GQ497940, EU977596, JX254652].  *Arthrobacter pigmenti* strain: LMG 22284 16S ribosomal RNA, partial sequence isolated from deteriorated mural paintings [NR_042250]. | 95  95 | KC535293 |
|  | 4.8% | B9-K21 | [626] | *Brachybacterium fresconis* and *Brachybacterium sacelli* partial 16S rRNA gene, strains [NR_025503, NR_025504] isolated from deteriorated parts of a medieval wall painting of the chapel of Herberstein Castle (Austria). | 99 | KC535285 |
|  | 2.4% | B9-K32 | [626] | *Brachybacterium zhongshanense* strain JB 16S ribosomal RNA, partial sequence [NR_044103], a cellulose-decomposing bacterium. | 98 | KC535291 |
|  | 2.4% | B9-K24 | [622] | *Cellulomonas* sp. d20 partial 16S rRNA gene, strain d20 [AJ298927]. | 96 | KC535286 |
|  | 4.8% | B9-K19 | [601] | *Kocuria* spp. [KC534176, JX949825, AB753819]. | 83 | KC535284 |
|  | 2.4% | B9-K30 | [507] | Uncultured bacterium clone ncd1290g07c1 [JF095540] associated with disease flares and treatment in children with atopic dermatitis.  *Ruania albidiflava* strain AS 4.3142 [NR_043736]. | 99  97 | KC535290 |
|  | 2.4% | B9-K47 | [507] | Uncultured bacterium clone ncd1290g07c1 [JF095540] associated with disease flares and treatment in children with atopic dermatitis.  *Ruania albidiflava* strain AS 4.3142 [NR_043736]. | 98  96 | KC535294 |
|  | 7.3% | B9-K27 | [630] | Uncultured bacterium clone ncd1290g07c1 [JF095540] associated with disease flares and treatment in children with atopic dermatitis.  *Ruania albidiflava* strain AS 4.3142 [NR_043736]. | 99  97 | KC535288 |
|  | 7.3% | B9-K29 | [631] | Uncultured bacterium clone ncd1290g07c1 [JF095540] associated with disease flares and treatment in children with atopic dermatitis.  *Ruania albidiflava* strain AS 4.3142 [NR_043736]. | 99  97 | KC535289 |
| **Sample C4: clothes** | | | | | | |
| *Proteobacteria*  Gammaproteobact.  (89.1%) | 45.6% | B2-K7 | [644] | Uncultured bacterial clones ribosomal RNA gene, partial sequence [EU735712, EU735714] from groundwater aquifer.  *Halomonas phoceae* strain CCUG 5096 16S ribosomal RNA gene, partial sequence [AY922995] isolated from human samples. | 99  98 | KC535202 |
|  | 6.5% | B2-K10 | [644] | *Halomonas muralis* partial 16S rRNA gene [AJ320531, AJ320532, NR_025486] isolated from microbial biofilms colonizing the walls and murals of the Catherine Chapel (Herberstein Castle, Austria). | 99 | KC535204 |
|  | 2.2% | B2-K25 | [644] | *Halomonas muralis* partial 16S rRNA gene [AJ320531, AJ320532, NR_025486] isolated from microbial biofilms colonizing the walls and murals of the Catherine Chapel (Herberstein Castle, Austria). | 99 | KC535209 |
|  | 2.2% | B2-K12 | [642] | Uncultured gamma proteobacteria clones 16S ribosomal RNA gene, partial sequence [JN408948, JN408987] in rhizosphere soil.  *Halomonas* spp. partial 16S rRNA gene, strain IB-O18 [AM490136, JQ028721, GU217708]. | 99  98 | KC535205 |
|  | 2.2% | B2-K39 | [644] | Uncultured gamma proteobacteria clones 16S ribosomal RNA gene, partial sequence [JN408970, JN408892] in rhizosphere soil.  *Halomonas muralis* partial 16S rRNA gene [AJ320531, AJ320532, NR_025486] isolated from microbial biofilms colonizing the walls and murals of the Catherine Chapel (Herberstein Castle, Austria). | 99  98 | KC535212 |
|  | 4.3% | B2-K40 | [644] | Uncultured gamma proteobacteria clones 16S ribosomal RNA gene, partial sequence [JN408970, JN408948] in rhizosphere soil.  *Halomonas muralis* partial 16S rRNA gene [AJ320531, AJ320532, NR_025486] isolated from microbial biofilms colonizing the walls and murals of the Catherine Chapel (Herberstein Castle, Austria). | 98  98 | KC535213 |
|  | 8.7% | B2-K44 | [644] | Uncultured gamma proteobacteria clones 16S ribosomal RNA gene, partial sequence [JN408970, JN408892] in rhizosphere soil.  *Halomonas muralis* partial 16S rRNA gene, strain MO-15 [HE964771] isolated from marine environment degrading textile dye at high salt concentration.  *Halomonas lutea* strain YIM 91125 16S ribosomal RNA, partial Sequence [NR_044350]. | 99  98  98 | KC535215 |
|  | 6.5% | B2-K8 | [644] | *Chromohalobacter* spp.16S ribosomal RNA gene, partial sequence [JN196510, JN196511, JN196512] in hypersaline environments.  *Chromohalobacter nigrandesensis* strain Taxon 4 16S ribosomal RNA, partial sequence [NR_042011] a moderately halophilic, Gram-negative bacterium isolated from Lake Tebenquiche, Atacama Saltern, Chile. | 98  98 | KC535203 |
|  | 2.2% | B2-K16 | [644] | *Chromohalobacter canadensis* strain RS93 16S ribosomal RNA gene, partial sequence [JQ638653] from potassium salt mine.  *Chromohalobacter beijerinckii* strain: NBRC 10304116S ribosomal RNA gene, partial sequence [AB681921].  *Chromohalobacter japonicus* strain 43 16S ribosomal RNA, partial sequence [NR_040965] from salty soils. | 99  99  99 | KC535206 |
|  | 6.5% | B2-K28 | [645] | *Chromohalobacter* spp. 16S ribosomal RNA gene, partial sequence [HQ683734, HQ683729].  *Chromohalobacter canadensis* strain RS93 16S ribosomal RNA gene, partial sequence [JQ638653] from potassium salt mine.  *Chromohalobacter beijerinckii* strain: NBRC 10304116S ribosomal RNA gene, partial sequence [AB681921].  *Chromohalobacter japonicus* strain 43 16S ribosomal RNA, partial sequence [NR_040965] from salty soils. | 91  91  91  91 | KC535210 |
|  | 2.2% | B2-K19 | [645] | Uncultured bacterial clones 16S ribosomal RNA gene, partial sequences [JX865182, JX865115], microbial communities in the Gulf of Trieste.  *Salinisphaera* spp. p 16S ribosomal RNA gene, partial sequence [GU452539, JF775510, JN020587, HM137559] | 98  98 | KC535207 |
| *Actinobacteria*  (8.7%) | 2.2% | B2-K23 | [629] | Uncultured bacterium clone nbw113d01c1 16S ribosomal RNA gene, partial sequence [GQ008081] from the human skin microbiome.  *Pseudonocardia* spp. p 16S ribosomal RNA gene, partial sequence [GU574091, GU574092] from mould-colonized water-damaged building material. | 99  99 | KC535208 |
|  | 2.2% | B2-K36 | [641] | *Jiangella* sp. 13658J 16S ribosomal RNA gene, partial sequence [EU741189].  *Jiangella alkaliphila* strain YD8-87 = JBRI 2008 16S ribosomal RNA, partial sequence [NR_042592] isolated from a cave. | 98  97 | KC535211 |
|  | 2.2% | B2-K43 | [628] | *Jiangella* spp. 16S ribosomal RNA gene, partial sequence [GU574118, GU574120, GU574069] from mould-colonized water-damaged building material. | 98 | KC535214 |
|  | 2.2% | B2-K46 | [636] | *Streptomonospora* spp. 16S ribosomal RNA gene, partial sequence [JN633949, JN633954] from marine caves.  *Streptomonospora halophila* strain YIM 91355 16S ribosomal RNA, partial sequence [NR_044207], a halophilic actinomycete isolated from a hypersaline soil. | 91  91 | KC535216 |
| *Bacteroidetes*  (2.2%) | 2.2% | B2-K50 | [636] | *Bacteroidetes* bacteria 16S ribosomal RNA gene, partial sequence [JQ923476, JQ923475] *Alifodinibius roseus* gen. nov., sp. nov., and *Alifodinibius sediminis* sp. nov., isolated from salt mine sample. | 99 | KC535217 |
