# Supplementary material for: Microbial survey of the mummies from the Capuchin Catacombs of Palermo, Italy: biodeterioration risk and contamination of the indoor air
Source: FEMS Microbiol Ecol. 2013 Jul 9;86(2):341–56. doi: 10.1111/1574-6941.12165 (PMC3916889; doi:10.1111/1574-6941.12165)
Supplement: Table S2 — Phylogenetic affiliations of the archaeal partial 16S rRNA coding sequences detected in the Capuchin Catacombs, Palermo. [file fem0086-0341-sd3.docx]

**Table S2.** Phylogenetic affiliations of the archaeal partial 16S rRNA coding sequences detected in the Capuchin Catacombs, Palermo.

| **Clone abundance**  **(%)** | **Selectedclone** | **Length**  **[bp]** | Closest identified phylogenetic relatives [EMBL accession numbers] | **Similar.**  **(%)** | **Accession**  **number** |
| --- | --- | --- | --- | --- | --- |
| **Sample**  **C1+F6: Bones** | | | | | |
| 18.2% | A3-K5 | [489] | Uncultured *Halobacterium* clone, partial 16S rRNA gene [FN433760] detected on the extreme environment presented by salt-attacked monuments.  Uncultured *Halobacterium* clone partial 16S rRNA gene [AM159640], extreme halophilic microorganisms on mural paintings. | 95  95 | KC535258 |
| 3% | A3-K8 | [376] | Uncultured *Halococcus dombrowskii* clone K25, partial 16S rRNA gene, [AM159642], extreme halophilic microorganisms on mural paintings. | 99 | KC535259 |
| 3% | A3-K12 | [554] | Uncultured *Halobacterium* clones partial 16S rRNA gene [FN435879, FN433761] detected on the extreme environment presented by salt-attacked monuments.  *Halococcus dombrowskii* strain H4 16S ribosomal RNA, complete sequence [NR_028186], an archaeal isolate from a Permian alpine salt deposit. | 96  96 | KC535260 |
| 3% | A3-K20 | [538] | Uncultured *Halobacterium* clones partial 16S rRNA gene [FN435851] detected on the extreme environment presented by salt-attacked monuments.  *Halococcus* sp. BIGigoW09 partial 16S rRNA gene, strain BIGigoW09 [AM902587] isolates from rock salt, Austria. | 97  94 | KC535261 |
| 3% | A3-K24 | [555] | Uncultured *Halobacterium* clones, partial 16S rRNA gene [FN433760, FN435879] detected on the extreme environment presented by salt-attacked monuments.  *Halococcus* sp. BIGigoW09 partial 16S rRNA gene, strain BIGigoW09 [AM902587] isolates from rock salt, Austria. | 99  98 | KC535262 |
| 6.1% | A3-K39 | [554] | Uncultured *Halobacterium* clones partial 16S rRNA gene [FN435879, FN433761] detected on the extreme environment presented by salt-attacked monuments.  *Halococcus dombrowskii* strain H4 16S ribosomal RNA, complete sequence [NR_028186], an archaeal isolate from a Permian alpine salt deposit. | 99  99 | KC535263 |
| 3% | A3-K41 | [550] | Uncultured *Halobacterium* clones partial 16S rRNA gene [FN435879, FN433761] detected on the extreme environment presented by salt-attacked monuments.  *Halococcus dombrowskii* strain H4 16S ribosomal RNA, complete sequence [NR_028186], an archaeal isolate from a Permian alpine salt deposit. | 99  99 | KC535264 |
| 57.6% | A3-K45 | [555] | Uncultured *Halobacterium* clones partial 16S rRNA gene [FN435879, FN433761] detected on the extreme environment presented by salt-attacked monuments.  *Halococcus dombrowskii* strain H4 16S ribosomal RNA, complete sequence [NR_028186], an archaeal isolate from a Permian alpine salt deposit. | 99  99 | KC535265 |
| 3% | A3-K46 | [555] | Uncultured *Halobacterium* clones, partial 16S rRNA gene [FN433760, FN435879, FN433761] detected on the extreme environment presented by salt-attacked monuments.  *Halococcus dombrowskii* strain H4 16S ribosomal RNA, complete sequence [NR_028186], an archaeal isolate from a Permian alpine salt deposit. | 98  98 | KC535266 |
| **Sample W2: salt efflorescence** | | | | | |
| 4% | A7-K14 | [555] | Uncultured archaeon clones partial 16S rRNA gene [AJ291421, AJ291422] detected in deteriorated ancient wall paintings. | 98 | KC535267 |
| 64% | A7-K15 | [555] | Uncultured archaeon H6-K5 partial 16S rRNA gene [AJ291421] detected in deteriorated ancient wall paintings. | 99 | KC535268 |
| 8% | A7-K42 | [499] | Uncultured *Halobacterium* clone K14 partial 16S rRNA gene [AM159641], extreme halophilic microorganisms on mural paintings. | 96 | KC535269 |
| 16% | A7-K75 | [555] | Halophilic archaeon strains gene for 16S ribosomal RNA, partial sequence [AB588755, AB588757, AB588758], an extreme halophile of the order Halobacteriales.  *Halococcus salifodinae* 16S rRNA gene, strain BG2/2 [AJ131458], permo-triassic salt deposits. | 97  97 | KC535270 |
| 8% | A7-K89 | [556] | Uncultured *Halobacterium* clone K14 partial 16S rRNA gene [AM159641], extreme halophilic microorganisms on mural paintings. | 97 | KC535271 |
| **Sample C3 : stuffing material** | | | | | |
| 4.9% | A9-K19 | [515] | Uncultured *Halobacterium* clone K14 partial 16S rRNA gene [AM159641 ], extreme halophilic microorganisms on mural paintings.  Uncultured *Halobacterium* clones partial 16S rRNA gene [FN433757, FN435859] detected on the extreme  environment presented by salt-attacked monuments. | 95  95 | KC535272 |
| 12.2% | A9-K24 | [495] | Uncultured *Halobacterium* clone K14 partial 16S rRNA gene [AM159641], extreme halophilic microorganisms on mural paintings. | 98 | KC535273 |
| 2.4% | A9-K30 | [555] | Uncultured archaeon clone 362 16S ribosomal RNA gene, partial sequence [EF188372] detected in white colonizations in Altamira Cave. | 98 | KC535274 |
| 4.9% | A9-K39 | [555] | Uncultured Halobacterium clone K14 partial 16S rRNA gene [AM159641], extreme halophilic microorganisms on  mural paintings.  Uncultured *Halobacterium* clones partial 16S rRNA gene [FN433757, FN435859] detected on the extreme environment presented by salt-attacked monuments. |  | KC535275 |
| 2.4% | A9-K41 | [555] | Uncultured archaeon clones partial 16S rRNA gene [AJ291421, AJ291422] detected in deteriorated ancient wall paintings. | 99 | KC535276 |
| 46.3% | A9-K51 | [555] | Uncultured archaeon H6-K5 partial 16S rRNA gene [AJ291421] detected in deteriorated ancient wall paintings. | 99 | KC535277 |
| 7.3% | A9-K57 | [556] | Halophilic archaeon strains gene for 16S ribosomal RNA, partial sequence [AB588755, AB588757, AB588758], an extreme halophile in the order Halobacteriales.  Uncultured *Halococcus salifodinae* partial 16S rRNA gene, clone K12 [AM159639], extreme halophilic microorganisms on mural paintings. | 98  98 | KC535278 |
| 12.2 | A9-K63 | [555] | Uncultured archaeon H6-K5 partial 16S rRNA gene [AJ291421] detected in deteriorated ancient wall paintings.  Uncultured *Halobacterium* clone K14 partial 16S rRNA gene [AM159641], extreme halophilic microorganisms on mural paintings. | 97  97 | KC535279 |
| 2.4% | A9-K64 | [555] | Uncultured archaeon H6-K5 partial 16S rRNA gene [AJ291421] detected in deteriorated ancient wall paintings. | 98 | KC535280 |
| 4.9% | A9-K98 | [556] | Uncultured *Halobacterium* clone K14 partial 16S rRNA gene [AM159641], extreme halophilic microorganisms on mural paintings.  Uncultured *Halobacterium* clones partial 16S rRNA gene [FN433757, FN435859] detected on the extreme environment presented by salt-attacked monuments. | 97  97 | KC535281 |
