# Supplementary material for: Microbial survey of the mummies from the Capuchin Catacombs of Palermo, Italy: biodeterioration risk and contamination of the indoor air
Source: FEMS Microbiol Ecol. 2013 Jul 9;86(2):341–56. doi: 10.1111/1574-6941.12165 (PMC3916889; doi:10.1111/1574-6941.12165)
Supplement: Table S3 — Phylogenetic affiliations of the fungal ITS coding sequences obtained from human remains held in the Capuchin Catacombs, Palermo. [file fem0086-0341-sd4.docx]

**Table S3.** Phylogenetic affiliations of the fungal ITS coding sequences obtained from human remains held in the Capuchin Catacombs, Palermo.

| **Clone abundance (%)** | **Selected clone** | **Length**  **[bp]** | Closest identified phylogenetic relatives [EMBL accession numbers] | **Similar.**  **(%)** | **Accession**  **number** |
| --- | --- | --- | --- | --- | --- |
| **Sample**  **M2+N2: skin** | | | | | |
| 10.2% | F5-K24 | [401] | Ascomycota sp. SAB-A1B-T [JX869562] isolated from textile materials inside a crypt.  *Sagenomella* sp. HMH-2007a [EU140821] responsible for bilateral keratitis. | 93  92 | KC535139 |
| 89.8% | F5-K49 | [576] | *Phialosimplex chlamydosporus* strain UAMH 10961 [GQ169326] associated with infections in dogs and having phylogenetic affinity to the Trichocomaceae. | 90 | KC535140 |
| **Sample P1: muscle** | | | | | |
| 72.9% | F1-K1 | [574] | *Phialosimplex chlamydosporus* strain UAMH 10961 [GQ169326], a new anamorphic genus associated with infections in dogs and having phylogenetic affinity to the Trichocomaceae.  *Phialosimplex caninus* strains [GQ169313, GQ169315, GQ169316, GQ169317], anamorphic genus associated with infections in dogs and having phylogenetic affinity to the Trichocomaceae. | 91  91 | KC535119 |
| 2.1% | F1-K28 | [562] | *Phialosimplex chlamydosporus* strain UAMH 10961 [GQ169326], a new anamorphic genus associated with infections in dogs and having phylogenetic affinity to the Trichocomaceae.  *Phialosimplex caninus* strains [GQ169313, GQ169315, GQ169316, GQ169317], anamorphic genus associated with infections in dogs and having phylogenetic affinity to the Trichocomaceae. | 91  91 | KC535124 |
| 8.3% | F1-K30 | [575] | *Phialosimplex chlamydosporus* strain UAMH 10961 [GQ169326], a new anamorphic genus associated with infections in dogs and having phylogenetic affinity to the Trichocomaceae.  *Phialosimplex caninus* strains [GQ169313, GQ169315, GQ169316, GQ169317], anamorphic genus associated with infections in dogs and having phylogenetic affinity to the Trichocomaceae. | 91  90 | KC535125 |
| 2.1% | F1-K4 | [574] | *Phialosimplex chlamydosporus* strain UAMH 10961 [GQ169326], a new anamorphic genus associated with infections in dogs and having phylogenetic affinity to the Trichocomaceae.  *Phialosimplex caninus* strains [GQ169313, GQ169315, GQ169316, GQ169317], anamorphic genus associated with infections in dogs and having phylogenetic affinity to the Trichocomaceae. | 91  91 | KC535120 |
| 10.4% | F1-K5 | [362] | *Sagenomella* sp. ASR-61, partial sequence [GU973651]. | 93 | KC535121 |
| 2.1% | F1-K25 | [348] | Uncultured fungal clones, partial sequence [GU053993, GU053994] in continental and marine air. | 98 | KC535123 |
| 2.1% | F1-K11 | [608] | Uncultured fungus [FR682462] settled dust of moisture- damaged buildings. | 98 | KC535122 |
| **Sample**  **M1+N3: hair** | | | | | |
| 17.8% | F4-K4 | [574] | *Phialosimplex chlamydosporus* strain UAMH 10961 [GQ169326] associated with infections in dogs and having phylogenetic affinity to the Trichocomaceae. | 91 | KC535127 |
| 2.2% | F4-K30 | [491] | *Phialosimplex chlamydosporus* strain UAMH 10961 [GQ169326] associated with infections in dogs and having phylogenetic affinity to the Trichocomaceae. | 92 | KC535133 |
| 2.2% | F4-K40 | [574] | *Phialosimplex chlamydosporus* strain UAMH 10961 [GQ169326] associated with infections in dogs and having phylogenetic affinity to the Trichocomaceae. | 91 | KC535136 |
| 15.6% | F4-K48 | [582] | Uncultured fungal clones [JQ410077, JQ410084] at archaeological excavation site of catacombs of Xizhou Dynasty, China.  *Phialosimplex chlamydosporus* strain UAMH 10961 [GQ169326] associated with infections in dogs and having phylogenetic affinity to the Trichocomaceae. | 97  93 | KC535137 |
| 20% | F4-K14 | [499] | Uncultured fungus clone T5-F113 [JF812152] associated to the decayed medieval stained window glasses of two Catalonian churches.  *Penidiella venezuelensis* strain CBS 106.75 [EU019278]. | 99  95 | KC535128 |
| 8.9% | F4-K24 | [533] | Uncultured fungus clone T5-F113 [JF812152] associated to the decayed medieval stained window glasses of two Catalonian churches.  *Penidiella venezuelensis* strain CBS 106.75 [EU019278]. | 98  96 | KC535129 |
| 2.2% | F4-K25 | [544] | Uncultured fungus clone T5-F113 [JF812152] associated to the decayed medieval stained window glasses of two Catalonian churches.  *Penidiella venezuelensis* strain CBS 106.75 [EU019278]. | 98  96 | KC535130 |
| 2.2% | F4-K26 | [530] | Uncultured fungus clone T5-F113 [JF812152] associated to the decayed medieval stained window glasses of two Catalonian churches.  *Penidiella venezuelensis* strain CBS 106.75 [EU019278]. | 100  94 | KC535131 |
| 8.9% | F4-K50 | [533] | *Penidiella venezuelensis* strain CBS 106.75 [EU019278]. | 99 | KC535138 |
| 8.9% | F4-K28 | [491] | *Eupenicillium lassenii* strain NRRL 5272 [AF033430] isolated from the conidial heads of *Aspergillus* species. | 89 | KC535132 |
| 2.2% | F4-K39 | [533] | *Aspergillus vitricola* isolates [EF652046, JX156358]. | 95 | KC535135 |
| 4.4% | F4-K2 | [576] | Fungal sp. VKM FW-2422 FJ609298 from permafrost.  *Pleosporales* sp. r292 [HQ649943] associated with roots of halophytic and non-halophytic plant species vary differentially along a salinity gradient. | 94  92 | KC535126 |
| 4.4% | F4-K34 | [604] | *Rhizopus oryzae* strains [AY803930, AY803931], human pathogenic, a major mucormycosis agent. | 99 | KC535134 |
| **Sample**  **C1+F6: bones** | | | | | |
| 79.1% | F3-K1 | [483] | *Penicillium radicum* strains [AY256855, AB457007]. | 100 | KC535111 |
| 8.3% | F3-K10 | [583] | *Gymnoascus petalosporus*  [AB361639]. | 86 | KC535113 |
| 2.1% | F3-K33 | [600] | *Gymnoascus petalosporus*  [AB361639]. | 84 | KC535117 |
| 2.1% | F3-K6 | [600] | *Gymnoascus petalosporus*  [AB361639]. | 84 | KC535112 |
| 2.1% | F3-K25 | [600] | *Gymnoascus petalosporus*  [AB361639]. | 84 | KC535116 |
| 2.1% | F3-K14 | [599] | *Gymnoascus petalosporus*  [AB361639]. | 88 | KC535114 |
| 2.1% | F3-K15 | [499] | *Gymnoascus petalosporus*  [AB361639]. | 88 | KC535115 |
| 2.1% | F3-K41 | [546] | *Gymnoascus petalosporus*  [AB361639]. | 87 | KC535118 |
| **Sample C3 : stuffing material** | | | | | |
| 85.7% | F9-K27 | [578] | *Stephanoascus ciferrii* strain CBS 5295 gene, partial sequence [AY493435]. | 85 | KC535144 |
| 4.1% | F9-K2 | [578] | *Stephanoascus ciferrii* strain CBS 5295 gene, partial sequence [AY493435]. | 85 | KC535141 |
| 2% | F9-K49 | [578] | *Stephanoascus ciferrii* strain CBS 5295 gene, partial sequence [AY493435]. | 85 | KC535146 |
| 4.1% | F9-K24 | [520] | Uncultured *Pichia* clone AWW2 [JX448363], fungal diversity in agarwood. | 85 | KC535143 |
| 2% | F9-K19 | [576] | *Phialosimplex chlamydosporus* strain UAMH 10961 [GQ169326] associated with infections in dogs and having  phylogenetic affinity to the Trichocomaceae.  Uncultured fungus clone SXF24 [JQ410077], fungi at archaeological excavation site of catacombs of Xizhou Dynasty, China. | 91  91 | KC535142 |
| 2% | F9-K28 | [559] | Ascomycota sp. SAB-A1B-T [JX869562] isolated from textile materials inside a crypt.  *Phialosimplex chlamydosporus* strain UAMH 10961 [GQ169326] associated with infections in dogs and having phylogenetic affinity to the Trichocomaceae. | 96  94 | KC535145 |
| **Sample C4: clothes** | | | | | |
| 11.2% | F2-K5 | [540] | Uncultured fungus clone SXF3 [JQ410056, JQ410060] from an archaeological excavation site.  *Acremonium nepalense* isolate Ppf33 [GU586837]. | 97  94 | KC535100 |
| 11.2% | F2-K20 | [354] | *Acremonium charticola* culture-collection UOA/HCPF<GRC>:14413 [KC253940], common and emerging mould pathogens in Greece. | 99 | KC535103 |
| 6.6% | F2-K23 | [578] | Uncultured fungus [FR682361] settled dust of moisture-damaged buildings.  Acremonium sp. [EF042103]. | 98  95 | KC535105 |
| 6.6% | F2-K28 | [570] | Uncultured fungus [FR682361] settled dust of moisture-damaged buildings.  *Acremonium* spp. [EF042103]. | 97  96 | KC535108 |
| 4.4% | F2-K48 | [507] | *Acremonium charticola* culture-collection UOA/HCPF<GRC>:14413 [KC253940], common and emerging mould pathogens in Greece. | 97 | KC535109 |
| 2.2% | F2-K49 | [419] | Uncultured fungus [FR682361] settled dust of moisture-damaged buildings.  *Acremonium* spp. [EF042103]. | 95  94 | KC535110 |
| 26.7% | F2-K2 | [382] | *Nectria balansae* strain AR4446, partial sequence [JN995620].  *Nectria sinensis* strain 7145 partial sequence [HM054139]. | 94  94 | KC535099 |
| 6.6% | F2-K33 | [349] | *Nectria balansae* strain AR4446 [JN995620].  *Nectria sinensis* strain 7145 [HM054139]. | 92  92 | KC535107 |
| 2.2% | F2-K24 | [550] | *Nectria* spp. [HM484546, HM484545]. | 91 | KC535106 |
| 17.8% | F2-K15 | [561] | *Phialophora* spp. [FJ489612, JQ766442, JQ766444] from human skin and possible role in human infection. | 82 | KC535102 |
| 2.2% | F2-K8 | [518] | *Phialophora* sp. [HQ608107]. | 85 | KC535101 |
| 2.2% | F2-K22 | [552] | *Devriesia queenslandica* culture-collection CPC:17306 [JF951148]. | 93 | KC535104 |
